# Classification of direct threats to the conservation of ecosystems and species 4.0

**DOI:** 10.1111/cobi.14434

**Published:** 2024-12-31

**Authors:** Nick Salafsky, Claire Relton, Bruce E. Young, Philippe Lamarre, Monika Böhm, Maxime Chénier, Erica Cochrane, Mark Dionne, Kevin K. He, Craig Hilton‐Taylor, Charles Latrémouille, John Morrison, Calla V. Raymond, Mary Seddon, Varsha Suresh

**Affiliations:** ^1^ Foundations of Success, Conservation Measures Partnership (CMP) & IUCN World Commission on Protected Areas Bethesda Maryland USA; ^2^ Foundations of Success & CMP Cape Town South Africa; ^3^ NatureServe & IUCN Species Survival Commission (SSC) Arlington Virginia USA; ^4^ Ministère de l'Environnement, de la Lutte contre les changements climatiques de la Faune et des Parcs (MELCCFP) Québec Québec Canada; ^5^ Indianapolis Zoo & IUCN SSC Indianapolis Indiana USA; ^6^ Environment & Climate Change Canada Québec Québec Canada; ^7^ International Crane Foundation & CMP Baraboo Wisconsin USA; ^8^ Pew Charitable Trusts & CMP Washington, DC USA; ^9^ IUCN Cambridge UK; ^10^ Independent Consultant & CMP Montreal Québec Canada; ^11^ World Wildlife Fund & Conservation Coaches Network Washington, DC USA; ^12^ Environment & Climate Change Canada Canadian Wildlife Service Gatineau Québec Canada; ^13^ IUCN SSC Devon UK; ^14^ Foundations of Success & CMP New York New York USA

**Keywords:** classification scheme, Conservation Measures Partnership, conservation science, conservation threat, IUCN Red List, pressure, Alianza para las Medidas de Conservación, amenaza a la conservación, ciencias de la conservación, esquema de clasificación, Lista Roja UICN, presión

## Abstract

Identifying and assessing the magnitude of direct threats to ecosystems and species are critical steps to prioritizing, planning, implementing, and assessing conservation actions. Just as medical clinicians and researchers need a standard way to talk about human diseases, conservation practitioners and scientists need a common and comprehensive language to talk about the threats they are facing to facilitate joint action, evaluation, and learning. To meet this need, in 2008 the IUCN Species Survival Commission and the Conservation Measures Partnership produced the first version of a common threats classification with the understanding that it would be periodically updated to take into account new information and learning. We present version 4.0 of this classification. For this latest update, we reviewed existing versions and derivatives of the original classification, over 1000 citations of the classification, threats data from over 2900 real‐world conservation projects, and comments from many users. Based on our findings, we made changes to the threats classification scheme, including addition of a level 0 threat class, refinement of levels 1 and 2 threat categories, and addition of the threat “Fencing & walls” to level 2. Also added were level 3 threat types and modifiers that provide a more detailed description of different types of direct threats and thus allow users to fine‐tune analyses and actions. The update also clarifies how to treat various stressors, including natural disaster events and climate change. As a result of these changes, we revised the formal definition of direct threats. They include human actions that are the direct cause of ecosystem or species‐population degradation and loss, such as agriculture, transport, natural resource use, and ecosystem management. They also include ultimate stressors in natural systems whose dynamics have been altered by the effects of current or historical human actions, such as invasive or problematic native species, pollution, natural disasters, and climate change.

## Abstract (es)

Clasificación de las amenazas directas a la conservación de ecosistemas y especies 4.0

**Resumen:**

La identificación y evaluación de la magnitud de las amenazas directas a los ecosistemas y las especies son fundamentales para priorizar, planificar, ejecutar y evaluar las acciones de conservación. Así como los médicos e investigadores necesitan una forma estándar para hablar de las enfermedades humanas, los profesionales y científicos de la conservación necesitan un lenguaje global e integral para hablar de las amenazas a las que se enfrentan y facilitar así la acción conjunta, la evaluación y el aprendizaje. Como respuesta a esta necesidad, la Comisión de Supervivencia de Especies de la UICN y la Alianza para las Medidas de Conservación elaboraron la primera versión de una clasificación común de amenazas en 2008, con el entendimiento de que se actualizaría periódicamente para considerar la información y el aprendizaje más recientes. Presentamos la versión 4.0 de esta clasificación, para la cual revisamos las versiones existentes y derivadas de la clasificación original, más de 1000 citas de la clasificación, datos sobre amenazas de más de 2900 proyectos de conservación en el mundo real y comentarios de varios usuarios. Con base en nuestras conclusiones, presentamos algunos cambios en el esquema de clasificación de las amenazas, como la adición de una clase de amenaza de nivel 0, el perfeccionamiento de las categorías de amenaza de los niveles 1 y 2, y la incorporación de la amenaza *cercas y muros* al nivel 2. También añadimos tipos de amenazas de nivel 3 y modificadores que proporcionan una descripción más detallada de los distintos tipos de amenazas directas y permiten así a los usuarios afinar los análisis y las acciones. La actualización también aclara cómo tratar diversas fuentes de estrés, entre ellas las catástrofes naturales y el cambio climático. Como resultado de estos cambios, replanteamos la definición formal de amenazas directas para que incluya las acciones humanas que son la causa directa de la degradación y pérdida de ecosistemas o poblaciones de especies, como la agricultura, el transporte, el uso de los recursos naturales y la gestión de los ecosistemas. También incluye los factores primordiales de estrés de los sistemas naturales cuya dinámica se ha visto alterada por los efectos de acciones humanas actuales o históricas, como las especies invasoras, especies nativas problemáticas, la contaminación, las catástrofes naturales y el cambio climático.

## INTRODUCTION

### Need for a standard classification of direct threats

**FIGURE 1:**
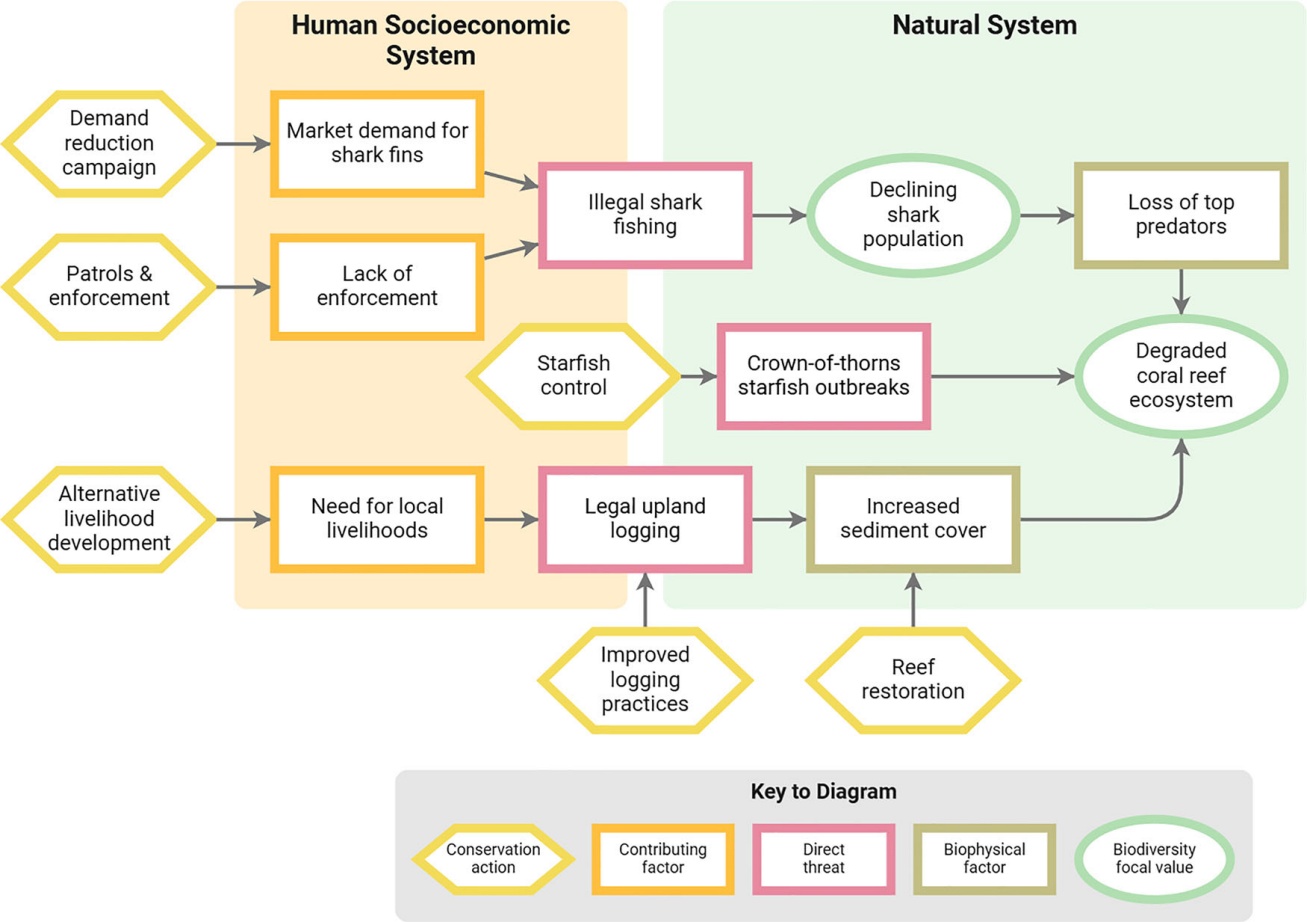
Example of a conservation situation showing 3 direct threats to ecosystems and species and their relation to other socioeconomic and natural system factors. See Table 1 for definitions of key terms.

Threats are one of the most important concepts in conservation practice. In the Open Standards for the Practice of Conservation 4.0 (CMP, 2020), direct threats (pink boxes in Figure 1) are defined as current or future human activities or altered natural phenomena that affect one or more ecosystems or species of interest (green ovals and collectively termed biodiversity focal values). Examples of direct threats include illegal fishing that affects a shark population, outbreaks of problematic native starfish that affect a coral reef ecosystem, legal logging that affects a downstream coral reef ecosystem, or climate change‐linked sea level rise that affects a mangrove ecosystem. Direct threats are the critical interface through which human socioeconomic systems affect and change natural systems and are the direct or indirect target of many conservation actions (see “DISCUSSION” for our formal revised definition of *direct threats* resulting from this work).

Identifying and assessing the magnitude of direct threats are critical steps to prioritizing, planning, implementing, and assessing conservation actions. For example, in International Union for Conservation of Nature (IUCN) Red List assessments, assessors are asked to identify the major threats affecting a species (IUCN, 2016). Threat assessments are also a critical step in the IUCN Green Status of Species assessment of species recovery and conservation impact (Akçakaya et al., 2018). Under the Open Standards for the Practice of Conservation (CMP, 2020), project and program teams are asked to identify and assess the magnitude of direct threats to their biodiversity focal values as a key part of developing their situation analyses and action plans. In commonly used assessments of protected area effectiveness, managers are asked to show progress in addressing direct threats as one major outcome metric, such as in the management effectiveness tracking tool (METT) (Stolton et al., 2021) and the IUCN–World Commission on Protected Areas (WCPA) Green List Standard (IUCN‐WCPA, 2017). Many national and subnational government agencies use threat identification and assessment in their work, including US state wildlife agencies (AFWA, 2012), the Chilean National Forest Corporation (CONAF, 2017), the Quebec Ministry of Forests, Fauna, and Parks (MFFP, 2021), and the Swedish Agency for Marine and Water Management (SwAM, 2021). And many researchers have used threat reduction as a key proxy‐dependent variable to assess the success of conservation actions, especially in instances where direct measurement of changes in the natural system and biodiversity focal values is infeasible and when time scales are long, including the threat reduction assessment (TRA) method (Salafsky & Margoluis, 1999) and the species threat abatement restoration (STAR) metric (Mair et al., 2021).

An essential foundation of any science is a standard lexicon—the equivalent of Linnaeus's classification system for living organisms in biology or Mendeleev's periodic table (Salafsky et al., 2008). Just as medical clinicians and researchers need a standard language to talk about human diseases, conservation practitioners and scientists need a common language to talk about the problems they are facing. At its simplest, imagine a central online database of conservation work in which one team of practitioners records that cattle are a threat, another team says livestock are a threat, and a third says grazing is a threat. In this scenario, these teams have no way of knowing if they are all grappling with the same problem and thus cannot take joint action or learn from one another. But if they agree on a common term—or at least link their specific terms to terms in a common vocabulary—they can have a productive dialogue and ultimately identify actions that work under different conditions to address the problem.

### History of this threat classification

To address this need for a common lexicon of direct threats, in the early 2000s, the IUCN and the Conservation Measures Partnership (CMP) independently began to develop classifications of direct threats as well as conservation actions (e.g., annex 5 in Hilton‐Taylor [2000], table 1 in Salafsky et al. [2002], CMP [2005], and IUCN [2005]). Recognizing that it is far more effective to have only one accepted global scheme, these groups then came together to produce version 1.0 of the unified threats classification (Salafsky et al., 2008). Because this classification became an international standard, it could not be changed too frequently. But it also needed to be updated periodically to clear up confusion and take into account new information and learning. To this end, the CMP convened a task force in 2013 to review and amend version 1.0. This work resulted in CMP's version 2.0 (CMP, 2015). The CMP's version 2.0 was reviewed by the IUCN Red List Committee but was not formally adopted for technical reasons. Instead, the IUCN produced a series of updated versions of its threat classification, culminating in version 3.3 (IUCN, 2022).

Starting in 2022, IUCN and CMP convened a joint task force to again consider revisions to the classification, the results of which are presented here. Because we are now re‐unifying the IUCN and CMP versions, we are producing this as version 4.0. This means that CMP skipped version 3.0. Although we believe version 4.0 substantially improves on previous versions and should be used where possible, the earlier versions are still valid classification schemes that can be used where relevant or where users cannot update previously classified data records.

## METHODS

To revise the classification, the task force reviewed how previous versions of the classification have been used and relevant classifications developed by other parties (e.g., Geyer et al., 2011), notably the classification developed and vetted by several Canadian institutions (MFFP, 2021). We also solicited and received more than 170 distinct comments and suggestions from IUCN threat classification users. The information gathered from these reviews formed the basis for an extensive review and amendment process by members of the task force to produce draft versions of the revised classification. The draft revisions were shared with numerous practitioners around the world for detailed comments and feedback, based on which we made additional revisions resulting in version 4.0.

Any classification system inherently reflects the mental models, biases, and needs of its creators; there is no one inherently right system. The task force used a slightly modified version of the criteria first established in Salafsky et al. (2008) to define the task force's ideal classification system. Classification entries had to be useful, simple, hierarchical, comprehensive, consistent, expandable, exclusive, and scalable. Specifically, to be useful, entries must be identified and organized in a way that makes sense to conservation practitioners. To be simple, language should be clear and understandable by all practitioners and examples should be provided. A hierarchical structure creates a logical grouping of threats that are related to one another to facilitate use of the classification and meaningful analyses at different levels. A comprehensive classification contains all possible threats at least at higher levels of the hierarchy (i.e., an *other* category is avoided). In a consistent classification, all entries at a given level are of the same type. In an expandable and exclusive classification, the list of threats can be modified to include newly discovered threats and any given threat can only belong to one cell in the hierarchy. Finally, to be scalable, the same threats should be applicable at individual sites or across a continent and to a single subpopulation or the global population of a species.

We used these criteria to assess and debate different options for each modification to the threats classification (e.g., simple terms, such as *hunting* and *fishing*, were preferred over more technically precise jargon, such as *consumptive use of animal species*). Recognizing that the task force was developing only the English language version of the classification, we tried where possible to select terms that are robust to translation issues to mitigate language‐based confusion. In a similar fashion, we also endeavored to select examples of threats from across the world.

To develop and check the utility of the revised classification, we analyzed specific threat factors from all real‐world projects and programs in the Miradi Software database of conservation projects as of late 2022. This analysis involved first reviewing the list of projects to remove duplicates and fictitious or teaching examples, resulting in 2943 projects that contained 32,271 distinct threat factors. Of these factors, 16,169 (50%) had been coded by their users to version 1.0 of the classification. We reviewed these factors noting whether users had coded them correctly to either the level 1 or level 2 threat level in version 1.0 and corrected them where necessary. We then coded the remaining threat factors to version 2.0 of the threats classification, noting any entries that could not be assigned to an existing level 2 category.

## RESULTS

Our results revealed that the threat classification is widely used by the conservation community. Anecdotal evidence shows that this classification system has been used to organize conservation thinking in many forums, such as the presentation of evidence for conservation actions on www.ConservationEvidence.com and the organization of major sessions at the 2016 IUCN World Conservation Congress.

Appendix S1 provides a summary of over 1000 peer‐reviewed literature citations of the 2008 published version of this classification (Salafsky et al., 2008). Some of these citations also refer to the companion conservation actions classification that was jointly published with the direct threats classification. The total number of citations has increased in recent years, indicative of rising interest in this topic. Based on a review of a subset of 570 of these papers, 330 of them were about specific conservation situations and 240 were about frameworks or methodologies for doing conservation work.

There were 3 common suggestions for improvements to earlier versions of the classification from a nonsystematic review of these papers and input from IUCN threat classification users. First, more detailed definitions and examples would be useful. Several papers (e.g., Battisti et al., 2015; Jono et al., 2012; MFFP, 2021) and numerous commenters called for more detailed definitions of the level 2 threat classifications and more comprehensive level 3 classifications to improve analyses of the causes of extinction risks and to better support identification of effective conservation measures. Second, a higher order organization was proposed. For example, Auld and Hobbs (2009) proposed organizing the classification into the following higher orders: ecosystem elimination, ecosystem degradation, and species decline and elimination. Third, several papers emphasized the need for a more detailed common classification system of threats to facilitate thorough situation analyses and improve conservation efforts.

**FIGURE 2:**
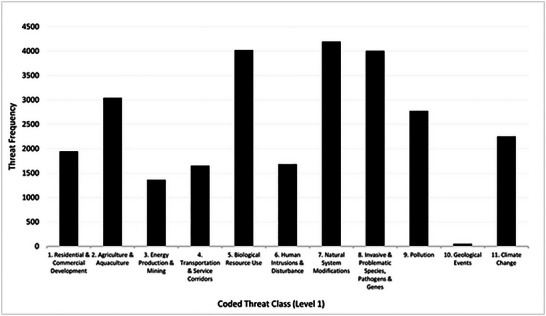
Frequency of threats from 2943 conservation projects coded to Conservation Measures Partnership Classification of Direct Threats 2.0 level 1 threats. Some projects had multiple threats.

Figure 2 and Table 2 provide a summary of the 32,271 threat factors we downloaded from 2943 real‐world projects in the Miradi Database. Appendix S2 provides the proportion of threats in each class that were judged to have been classified incorrectly. As discussed below in more detail, this analysis helped us assess the comprehensiveness of the threats classification.

**TABLE 1:** Definitions of key terms used in this paper to describe conservation situations.

Term	Definition^a^	Comment
Biodiversity focal value	Species or ecosystem that is the focus of one or more conservation actions	In CMP (2020), the primary term for this concept is conservation target. See Salafsky et al. (2024) for a discussion of why we are proposing this shift in terms.
Biophysical factor	Ecological or geophysical element in a natural system	Typically used to show the mechanism of how a direct threat affects a biodiversity focal value. In practice, it is usually sufficient to show these factors without differentiating them into stresses and stressors.
Stress	Biophysical factor that depicts a degraded attribute of a biodiversity focal value	Typically more proximate to the biodiversity focal values in a diagram
Stressor	Biophysical factor that causes stress to a biodiversity focal value	As discussed below, ultimate stressors can be considered direct threats.
Direct threat	Human action that is the direct cause of ecosystem or species‐population degradation and loss or an ultimate stressor in a natural system whose dynamics have been altered by the effects of current or historical human actions	This is the new definition developed in this article. It is synonymous with *pressure*, *source of stress*, and *direct driver*.
Contributing factor	Element of a human socioeconomic system that leads to a direct threat	Term is synonymous with *driver* and *root cause*.
Conservation action	Set of activities with a common focus that work together to achieve specific conservation goals and objectives along a theory of change	Term is synonymous with *intervention*.

^a^ Definitions adapted from CMP (2020), Salafsky and Margoluis (2021), and Salafsky et al. (2024). Examples in Figure 1.

**TABLE 2:** Frequency of threats from 2943 conservation projects coded to Conservation Measures Partnership Classification version 2.0 level 2 threat categories.

Level 1 threat order		
Level 2 threat classification	Threat frequency	%
1. Residential & commercial development	555^a^	1.7
1.1 Housing & urban areas	833	2.6
1.2 Commercial & industrial areas	275	0.9
1.3 Tourism & recreation areas	284	0.9
2. Agriculture & aquaculture	618^a^	1.9
2.1 Annual & perennial non‐timber crops	845	2.6
2.2 Wood & pulp plantations	159	0.5
2.3 Livestock farming & ranching	1071	3.3
2.4 Marine & freshwater aquaculture	351	1.1
3. Energy production & mining	74^a^	0.2
3.1 Oil & gas drilling	210	0.7
3.2 Mining & quarrying	904	2.8
3.3 Renewable energy	176	0.5
4. Transportation & service corridors	86^a^	0.3
4.1 Roads & railroads	1011	3.1
4.2 Utility & service lines	254	0.8
4.3 Shipping lanes	269	0.8
4.4 Flight paths	34	0.1
5. Biological resource use	283^a^	0.9
5.1 Hunting & collecting terrestrial animals	1206	3.7
5.2 Gathering terrestrial plants	182	0.6
5.3 Logging & wood harvesting	1200	3.7
5.4 Fishing & harvesting aquatic resources	1152	3.6
6. Human intrusions & disturbance	98^a^	0.3
6.1 Recreational activities	1339	4.1
6.2 War, civil unrest, & military exercises	78	0.2
6.3 Work & other activities	168	0.5
7. Natural system modifications	51^a^	0.2
7.1 Fire & fire suppression	1348	4.2
7.2 Dams & water management/use	2287	7.1
7.3 Other ecosystem modifications	475	1.5
7.4 Removing/reducing human maintenance	32	0.1
8. Invasive & problematic species, pathogens, & genes	338^a^	1.0
8.1 Invasive non‐native/alien plants & animals	2776	8.6
8.2 Problematic native plants & animals	351	1.1
8.3 Introduced genetic material	37	0.1
8.4 Pathogens & microbes	505	1.6
9. Pollution	502^a^	1.6
9.1 Household sewage & urban waste water	520	1.6
9.2 Industrial & military effluents	486	1.5
9.3 Agricultural & forestry effluents	630	2.0
9.4 Garbage & solid waste	328	1.0
9.5 Airborne pollutants	192	0.6
9.6 Excess energy	117	0.4
10. Geological events	3^a^	0.01
10.1 Volcanoes	5	0.02
10.2 Earthquakes/tsunamis	25	0.08
10.3 Avalanches/landslides	18	0.06
11. Climate change	707^a^	2.2
11.1 Ecosystem encroachment	253	0.8
11.2 Changes in geochemical regimes	68	0.2
11.3 Changes in temperature regimes	376	1.2
11.4 Changes in precipitation & hydrological regimes	410	1.3
11.5 Severe/extreme weather events	436	1.4
Biophysical factor	2300	7.1
Contributing factor	2292	7.1
Dummy factor	427	1.3
Unknown	261	0.8
Total	32,271	100

^a^ Reported frequencies for these level 1 orders reflect entries that could only be coded to this level; they are not a summation of level 2 values.

An overview of version 4.0 of the IUCN–CMP Classification of Direct Threats to Ecosystems and Species is presented in Table 3. The complete classification is available in Appendices S3 and S4 and also in an online version that includes a tracked‐changes line‐by‐line comparison with earlier versions of the classification. Analogous to the hierarchy in the Linnean classification of living things, the classification contains level 0 threat classes, level 1 threat orders, level 2 threat categories, and level 3 and 4 types. (An example of level 3 and 4 types is available in Table 4.) These levels are used to organize the classification and to facilitate grouping and analysis of like threats. As discussed in more detail below, specific threats should be identified using their common name (e.g., *illegal shark fishing* instead of “5.4 Fishing, harvesting & controlling aquatic species” or *legal upland logging* instead of “5.3 Logging, harvesting & controlling trees”). The direct threats classification has undergone some fundamental changes from previous versions.

**TABLE 3:** High‐level summary of International Union for Conservation of Nature–Conservation Measures Partnership Classification of Direct Threats to Ecosystems and Species version 4.0.

Level				
0	1	2	Definition	Examples
A. Use of lands & waters			Human uses of land and water areas that have a substantial spatial footprint. includes effects from their construction (e.g., ecosystem conversion), ongoing use, and abandonment	
	1. Residential, commercial & recreation areas		Human settlements, industrial areas, and other nonagricultural land uses with a substantial footprint	
		1.1 Residential areas	Cities, towns, and settlements including nonhousing development typically integrated with housing	Urban areas, suburbs, villages, rural housing, vacation homes, shopping areas, offices, schools, hospitals, houses of worship
		1.2 Commercial & industrial areas	Factories and other commercial centers	Stand‐alone office parks, stand‐alone shopping centers, manufacturing plants, military bases, coal or nuclear power plants, sewage treatment plants, landfills, train yards, ports, airports, land reclamation for industrial development
		1.3 Recreation & tourism areas	Tourism and recreation sites with a substantial spatial footprint that are not fully integrated into municipal areas	Visitor facilities in parks, campgrounds, football or other sports fields, ski areas, golf courses, beach facilities, fishing piers, marinas, cultural tourist attractions, pilgrimage facilities, cemeteries
	2. Agriculture & aquaculture		Farming and ranching including agricultural expansion, intensification, or practices with a spatial footprint; includes tree plantations, mariculture, and aquaculture	
		2.1 Annual & perennial non‐timber crops	Crops planted for food, fodder, fiber, fuel, or other uses	Farms, household swidden plots, oil palm plantations, orchards, vineyards, mixed agroforestry systems, biofuel crops, haying practices that disturb nesting birds
		2.2 Wood & pulp plantations	Stands of trees planted for timber or fiber outside of natural forests, often with non‐native species	Teak, eucalyptus or acacia plantations, pulp or fiber plantations, firewood lots, Christmas tree farms
		2.3 Terrestrial animal farming, ranching & herding	Domestic terrestrial animals raised in one location on farmed or nonlocal resources (farming); also domestic or semidomesticated animals allowed to roam in seminatural areas (ranching) or the wild and supported by natural habitats (herding)	Cattle feed lots, dairy farms, cattle ranching, chicken farms, goat, camel, or yak herding, game farms, bee keeping
		2.4 Marine & freshwater aquaculture	Aquatic species raised for harvest in artificial water bodies (analogous to terrestrial farming), enclosures in natural waters (analogous to ranching), or unenclosed natural waters (analogous to herding)	Fish ponds on farms, shrimp production in artificial ponds, salmon production in pens in natural water, hatchery salmon, seeded shellfish beds, artificial algal beds
	3. Energy production & mining		Extraction of nonbiological resources, often widely dispersed across the land / seascape	
		3.1 Oil & gas exploration & extraction	Exploring for and extracting petroleum and other liquid hydrocarbons	Oil wells, hydraulic fracturing, deep sea natural gas drilling
		3.2 Mining & quarrying	Exploring for and developing and producing minerals and rocks	Coal mines, alluvial gold panning, gold mines, rock quarries, sand or salt mining, coral mining, deep sea nodules, guano harvesting
		3.3 Renewable energy	Exploring for and developing and producing renewable energy	Geothermal power production, solar farms, wind farms (including birds or bats flying into windmills), tidal farms
	4. Transportation, service, & security corridors		Linear infrastructure such as long, narrow service or transport corridors including the effects associated with their use (e.g., mortality from vehicle collisions, restriction of species movement)	
		4.1 Roads, trails & railroads	Transport on roadways and dedicated tracks	Highways, secondary roads, logging roads, hiking or biking trails, bridges & causeways, vehicle collisions with wildlife, public transport systems, railroads
		4.2 Utility & service lines	Transport of energy and resources	Electrical & phone wires, aqueducts, oil & gas pipelines, electrocution of wildlife on power lines
		4.3 Shipping lanes	Transport on and in freshwater and ocean waterways	Maintenance of shipping channels, canals, shipping lanes, ships running into whales, wakes from cargo ships
		4.4 Atmospheric & space activities	Air and space transport and other activities	Flight paths, jets impacting birds, commercial or military drones, tethered balloons
		4.5 Fencing & walls	Barriers to movement	Border walls, fences around farm fields, fences or noise barriers along roads, protected area fencing, disease control fencing
B. Use / management of species & ecosystems			Human uses of biotic resources and disturbance from human presence or management actions in natural systems	
	5. Biological resource use & control		Consumptive use of wild biological resources including deliberate and unintentional harvesting effects as well as persecution of specific species	
		5.1 Hunting, collecting & controlling terrestrial animals	Hunting or trapping terrestrial wild animals for commercial, recreation, subsistence, research, or cultural purposes, or killing them for persecution or control reasons; includes nonlethal animal product harvesting and accidental mortality and bycatch	Subsistence hunting, collection of feathers or skins used in traditional ceremonies, commercial wild meat hunting, trophy hunting, fur trapping, insect collecting, pet trade, honey or egg collection, persecution of snakes, culling of deer, killing of crop‐raiding animals
		5.2 Gathering, harvesting & controlling terrestrial plants & fungi	Harvesting plants, fungi, and other nontimber and nonanimal products for commercial, recreation, subsistence, research, or cultural purposes, or persecution or control reasons; includes nonlethal product harvesting and accidental mortality and bycatch	Gathering wild fruit, mushrooms, orchids, rattan, lichen, or herbs for traditional medicine, collecting forage for stall fed animals, nonwoody biomass harvesting, rubber or maple syrup tapping, control of host plants to combat timber diseases
		5.3 Logging, harvesting & controlling trees	Harvesting trees and other woody vegetation for timber, fiber, or fuel, including site preparation and other forestry management practices; includes nonlethal coppicing and accidental mortality and bycatch	Clear cutting of hardwoods, selective commercial logging of ironwood, pulp operations, woody biomass collection, fuel wood collection, charcoal production, coppicing, tree thinning
		5.4 Fishing, harvesting & controlling aquatic species	Harvesting aquatic wild animals or plants for commercial, recreation, subsistence, research, or cultural purposes, or persecution or control reasons; includes nonlethal product harvesting and accidental mortality and bycatch	Net fishing, hook and line fishing, trawling, blast fishing, spear fishing, shellfish harvesting, whaling, seal hunting, turtle egg collection, live coral collection, aquarium fish collection, seaweed collection, persecution of sharks, control of seals
	6. Human intrusions & disturbances		Human activities that alter, disturb, and destroy ecosystems and species associated with nonconsumptive uses of biological areas and resources	
		6.1 Recreational activities	People spending time in natural areas or traveling in vehicles outside of established transport corridors, usually for recreational reasons	Hikers, mountain bikes, horse riding, off‐road vehicles, motorboats, jet skis, snowmobiles, ultralight planes, temporary campsites, caving, rock climbing, dive boats, whale watching boats, birdwatchers, pets in recreational areas
		6.2 Conflict, civil unrest & security activities	Actions in natural areas by formal or paramilitary forces without a permanent footprint	Armed conflict, riots, military training exercises, border patrols, peace keeping activities, guerilla camps, abandoned land mines, defoliation, munitions testing
		6.3 Other human disturbances	People spending time in or traveling in natural environments for reasons other than recreation or conflict and security activities	Drug smuggling or human migration through natural areas, livestock rustling, household water collection, nonrecreational bathing in rivers, festivals, pilgrimages, species research, archeological research, vandalism
	7 Natural system management & modifications		Human actions that modify ecosystem structures, composition, or regimes, generally to deliberately improve human welfare or benefit certain species. this category includes both construction of permanent or long‐term structures and their operations as well as more transitory management practices	
		7.1 Fire & fire management	Management actions that either suppress or increase fire frequency or intensity	Fire suppression to protect homes, inappropriate fire management, building of fire breaks, escaped agricultural fires, arson, campfires, fires for hunting
		7.2 Dams & water management / use	Management actions that modify water levels, flows, and chemistry	Dam construction, dam operations, levees and dikes, channelization, highway culverts, adding drains to wetlands for mosquito control, removal of natural beaver dams, encouraging beaver dams, water catchment areas, snow fences, dew harvesting, surface water withdrawals, groundwater pumping, increased humidity from human water use, water desalination, artificial lakes, birds drowning in artificial reservoirs, water treatment plants, adding lime to acid lakes
		7.3 Earth & sediment management	Management actions that modify the geophysical environment or change sediment regimes	Dune stabilization, sediment fencing, shoreline armoring, beach groins, soil pollution remediation, mine reclamation, land reclamation, dredging (except for shipping lanes)
		7.4 Weather & climate management	Management actions that modify atmospheric structure and processes	Cloud seeding, frost prevention, iron fertilization in the ocean, releasing reflective particles in the atmosphere, Lagrange point shades, carbon capture (except for infrastructure)
		7.5 Biological system management	Management actions that modify biotic systems including conservation actions that may have detrimental impacts on other nontargeted species or ecosystems	Mowing grass, using cattle to mimic natural grazing processes, removal of snags from streams, gating caves, artificial reef creation, assisted migration, bird feeders, electric barriers to stop invasive fish passage
		7.6 Removing / reducing human management		Absence or reduction of current or historical management regimes important for maintaining desired key ecological attributes of ecosystems or species Includes regimes historically maintained by protected area staff, farmers and ranchers, indigenous peoples, private landowners, or any other resource manager
C. Additional sources of stress			Stressors in natural systems that have been altered by the effects of current or historical human actions	
	8. Invasive / other problematic species, genes & pathogens		Threats from non‐native and native plants, animals, pathogens, microbes, or genetic materials that have or are predicted to have harmful effects on biodiversity following their introduction, spread, or increase in abundance or virulence	
		8.1 Invasive non‐native / alien species	Harmful plants, animals, and other species not originally found in the ecosystems in question and directly or indirectly introduced and spread into it by human activities	Rats on islands, feral horses, nonferal household pets, feral household pets, zebra mussels, bamboo, introduction of species for biocontrol, stocking exotic fish, ballast water discharge
		8.2 Problematic native species	Harmful plants, animals, and other species that are originally found in the ecosystems in question, but have become out of balance or released directly or indirectly due to human activities	Overabundant native deer, algal blooms, insect outbreaks
		8.3 Introduced genetic material	Human caused introduction of natural or synthetic genes into species in natural ecosystems	Pesticide resistant crops, hatchery salmon breeding with wild fish, domestic cats breeding with wild cats, restoration projects using nonlocal seed stock, genetically modified insects for biocontrol, genetically modified trees, genetically modified salmon or shellfish
		8.4 Pathogens	Harmful native and non‐native agents that cause disease or illness to a host species, including bacteria, viruses, prions, fungi, and other microorganisms	Plague affecting rodents, chronic wasting disease affecting cervids, Dutch elm disease or chestnut blight, chytrid fungus affecting amphibians, sea star wasting disease
	9. Pollution		Introduction of non‐native or excess materials or energy from point and nonpoint sources	
		9.1 Water‐borne & other effluent pollution	Water‐borne and other liquid pollutants stemming from various human activities; includes effects of these pollutants on the sites where they are generated or applied and where they end up in the environment	Discharge from municipal waste treatment plants, leaking septic systems, untreated sewage, outhouses, fertilizers and pesticides from lawns and golf‐courses, toxic chemicals from factories, illegal dumping of chemicals, nutrient loading from fertilizer runoff, herbicide runoff, manure from feedlots, excess nutrients from aquaculture, oil spills from pipelines, leaching from mine tailings, arsenic from gold mining, leakage from fuel tanks, oil or sediment from roads, road salt, erosion from logging operations, toxic chemicals in dredged river sediments
		9.2 Garbage & solid waste	Rubbish and other solid materials including those that entangle wildlife	Municipal waste, manure from livestock operations, mining tailings, litter from cars, agricultural plastics in soil, flotsam & jetsam from boats, microplastics, ghost fishing gear, construction debris, lead from hunting
		9.3 Air‐borne pollutants	Atmospheric pollutants from point and nonpoint sources	Acid rain from industry, wind dispersion of pollutants or particulates from farm fields, dust from roads, garbage incineration, methane flairs, smog from vehicle emissions, smoke from forest fires, radioactive fallout
		9.4 Energy emissions	Inputs of heat, sound, light, or other wave energy that disturb species or ecosystems	Beach lights disorienting turtles, heated water from power plants, seismic oil exploration, noise from highways or airplanes, electromagnetic fields from cables, sonar from submarines that disturbs whales, recreational boating wakes
	10. Natural disasters		Potentially catastrophic natural disturbances that conservation practitioners may still need to consider, particularly when managing small or remnant species populations or ecosystems	
		10.1 Geological events	Specific geological events that have potentially catastrophic effects on vulnerable species and ecosystems	Volcanic eruptions, earthquakes, tsunamis, avalanches, landslides
		10.2 Severe weather events	Specific weather events that have potentially catastrophic effects on vulnerable species and ecosystems	Rain or wind storms, hurricanes, cyclones, typhoons, hail storms, blizzards, dust storms, floods
	11. Climate change		Change in climate patterns resulting from increased atmospheric greenhouse gasses	
		11.1 Changes in physical & chemical regimes	Broad‐scale changes in the abiotic conditions of ecosystems	Ocean acidification, shifting aquatic oxygen minimum zone, changes in salinity, changes in atmospheric CO_2_ affecting plant growth, loss of sediment, changes in ocean currents, changes in jet stream, changes in cloud cover
		11.2 Changes in temperature regimes	Broad‐scale changes in temperature mean, variability, seasonality and extremes, including changes in temperature extremes, increased average summer temperature, and decreased minimum winter or spring temperature	Heat waves, cold spells, freeze and thaw cycles, oceanic temperature changes, marine heat blobs, loss of snowpack or glaciers, melting of sea ice
		11.3 Changes in precipitation & hydrological regimes	Broad‐scale changes in precipitation mean, variability, seasonality, and extremes, including decreased or increased precipitation, changes in timing of precipitation, changes in form of precipitation (e.g., snow vs. rain), changes in evapotranspiration rates and hydrological cycles, and droughts and floods	Rainfall patterns, droughts, timing of rains, reduced snow accumulation, increased severity of floods, sea‐level rise, shrinkage or loss of lakes
	12. Unknown threats		Threats that cannot be identified	

*Note*: A complete version of this table is in Appendix S3 and at https://docs.google.com/spreadsheets/d/1yfm7ua9hQJpjycx6FYQJ6Jy5LA4bP0XP61EW3-sX8ZY.

**TABLE 4:** Example of new level 3 and 4 threat type classifications in the International Union for Conservation of Nature–Conservation Measures Partnership Classification of Direct Threats to Ecosystems and Species 4.0.

Level 2 threat	Level 3 type	Optional level 3 modifiers			Level 4 proposals
		Life cycle	Regulation	Intensity	
2. Agriculture & aquaculture					
2.1 Annual & perennial non‐timber crops	Shifting cultivation Annual fixed cropping systems Perennial nontimber cropping systems other *(describe)*	Establishment Ongoing operations Abandoned	Legal & planned/regulated Legal but not planned/regulated Illegal &not planned/regulated	Subsistence or artisanal Small holder Industrial	Specify the crop(s) being grown and the production system
2.2 Wood & pulp plantations	Timber Pulpwood Ornamental trees Other *(describe)*	Establishment Ongoing operations Abandoned	Legal & planned/regulated Legal but not planned/regulated Illegal & not planned/regulated	Subsistence or artisanal Small holder Industrial	Specify the trees being grown and the type of production system
2.3 Terrestrial animal farming, ranching, & herding	Farming Ranching Herding or nomadic Other *(describe)*	Establishment Ongoing operations Abandoned	Legal & planned/regulated Legal but not planned/regulated Illegal & not planned/regulated	Subsistence or artisanal Small holder Industrial	Specify the animal(s) being farmed or ranched and the type of production system
2.4 Marine & freshwater aquaculture	Aquaculture in artificial water bodies Aquaculture in enclosed natural waters Aquaculture in open natural waters Other *(describe)*	Establishment Ongoing operations Abandoned	Legal & planned/regulated Legal but not planned/regulated Illegal & not planned/regulated	Subsistence or artisanal Small holder Industrial	Specify the taxa being produced and the type of production system

*Note*: A complete version of this table is in Appendix S4 and at https://docs.google.com/spreadsheets/d/1yfm7ua9hQJpjycx6FYQJ6Jy5LA4bP0XP61EW3‐sX8ZY.

### New level 0 threat classes

We grouped the existing level 1 threat orders into 3 higher level classes that highlight the similarities among the threats in each of these classes: “A. Use of lands & waters,” “B. Use/management of species & ecosystems,” and “C. Additional sources of stress.”

### Revisions to level 1 threat names

We made only one substantive change to the level 1 threat orders: “Geological events” was demoted from a level 1 entry in version 2.0 to a level 2 entry in the new level 1 entry “10. Natural disasters.” We also revised some of the level 1 threat names to clarify what they include.

### Additions and mergers of level 2 threats

Changes to some of the level 2 threat categories were substantive. We added “4.5 Fencing & walls” as a new level 2 threat in the level 1 order “4. Transportation, service & security corridors.” In general, this category applies to long, linear, or extensive perimeter features that could greatly restrict the movement of species.

We separated version 2.0's “7.3 Other ecosystem modifications” into “7.3 Earth & sediment management,” “7.4 Weather & climate management,” and “7.5 Biological system management” so that this order now contains management of fire, water, earth, air, and biodiversity. This led to a renumbering of what is now “7.6 Removing/reducing human management.”

We merged version 2.0's “9.1 Household sewage & urban waste water,” “9.2 Industrial & military effluents,” and “9.3 Agricultural & forestry water‐borne pollution” under “9.1 Water‐borne & other effluent pollution” and specified the specific source and pollutant type at level 3. This led to renumbering of the other level 2 entries in this order.

We moved v‐ersion 2.0's “11.5 Severe/extreme weather events” to “10.2 Severe weather events” as a new level 2 threat in this order. We deleted version 2.0's “11.1 Ecosystem encroachment” and moved sea level rise into “11.3 Changes in precipitation & hydrological regimes.” This led to renumbering of the other level 2 entries in this order. Finally, we clarified revisions to the names, definitions, and expositions of other level 2 threats.

### Addition of systematic level 3 and 4 threat types

Perhaps the biggest change to version 2.0 is that we added new level 3 and 4 threat types (see Table 4 for an example; the complete level 3 and 4 classifications are in Appendix S4). This addition builds on the work initiated in Canada (MFFP, 2021) and provides a more specific articulation of the higher level threat. Although we have attempted to be comprehensive in listing level 3 threat types, it is probable that additional types will need to be added in the future and types will need to be refined and amended. Therefore, although one of our principles for an ideal classification was to be as comprehensive as possible, we necessarily included an “other (describe)” option as an interim type to enable the development of a comprehensive list in a future update.

We added new optional level 3 modifiers that can be applied to the types. These are tailored for each level 1 threat. From a database perspective, these can be thought of as tags that can be applied to a given threat. Most of these modifiers are designed so that their component entries are mutually exclusive, but in some cases, it may make sense to allow multiple tags to be applied to a given threat (e.g., the types of pollution in urban effluent). These instances are noted as being multiselect. As discussed in more detail below, these modifiers explicitly do not try to encompass components of threat magnitude.

For some threats, we also included guidance on developing more detailed level 4 types. These entries often reference an external classification system that can be applied to provide a more detailed breakdown of the threat types. Finally, we made some revisions to the specific (noncomprehensive) examples for each threat category. These should be regarded as specific examples of the generic level 3 threat types.

Overall, given that the level 3 and 4 classifications are new and will likely require additional modifications and adjustments as they are tested and used, we are releasing them as a beta version.

## DISCUSSION

Over the course of our work, our task force encountered different issues and challenges that warrant discussion.

### Use of the term *threat* versus *pressure* or *driver*

Since the term *threat* began being used in the conservation field 3 decades ago (McNeely et al., 1990; Salafsky & Margoluis, 1999), conservation practitioners have expressed concerns that its use can backfire. For example, imagine working to develop a conservation plan with a group of cattle ranchers who graze their livestock in the grasslands you are trying to conserve or with Indigenous people who have a long history of hunting a species of conservation interest. These actors often feel that it is inappropriate to have their actions labeled as *threats* and may even argue that their management actions have been key to conserving these resources over time and are therefore not threats. In some extreme cases, calling their actions *threats* has led to these groups refusing to participate in the conservation work. Concerns about the term *threat* have also been raised by Indigenous groups who argue that it imposes an artificial separation between human and natural systems.

To deal with these concerns, many conservation practitioners have taken to either substituting the synonyms *pressure* or *driver* for *threat* (which seems less offensive to these groups) or adding modifying adjectives to the threat name (e.g., unsustainable grazing, unregulated hunting) to indicate that the problem is caused by bad applications of the human activity in question. The term *pressure* also has the advantage of linking to the “pressure–state–response” and similar frameworks developed in the 1990s (OECD, 1993). *Pressure* also seems preferable to the other synonym, *source of stress*, which was historically used as a part of The Nature Conservancy's 5‐S Conservation Action Planning Approach (TNC, 2006), but is too jargony for most conservation practitioners. Likewise, the term *direct driver* is used by the Intergovernmental Science‐Policy Platform on Biodiversity and Ecosystem Services (IPBES, 2024), although their definition covers factors that cause positive as well as negative effects on biodiversity.

However, use of the terms *pressure* or *direct driver* can be viewed as employing a polite euphemism to mask a serious problem facing one's biodiversity focal values. Just as medical doctors must confront the *diseases* (as opposed to *ailments* or *conditions*) facing their patients, conservationists must think realistically about the threats they are dealing with. Conservation is fundamentally a problem‐oriented discipline. Furthermore, even seemingly well‐managed grazing regimes can be a threat to some elements of biodiversity in the system. If current human actions truly do not intersect with and harm biodiversity of interest (e.g., organic farming on lands that have been cultivated for decades), then these actions should not be designated as threats in the first place.

Given the widespread adoption of the term *direct threat* across the conservation community (e.g., CMP, 2020; IUCN, 2016; IUCN & WCPA, 2017), we chose to retain this term as reflected in the title of this classification. If use of this term is not appropriate in a given situation, practitioners can substitute *pressure*, *direct driver*, or any other term as they see fit.

### Use of the classification to tag specific threats and aggregate them for analysis

In our review of uses of this classification, we noticed that some conservation practitioners have been too literal in using the entries to describe threats. For example, in Figure 1, their direct threat boxes might contain “*5.4 Fishing, harvesting & controlling aquatic species*” instead of *illegal shark fishing* and “*5.3 Logging, harvesting & controlling trees*” instead of *legal upland logging*. In doing so, they end up making their analyses more generic and less informative. A better approach is to treat the entries in the classification as scientific nomenclature categories that can be used to accompany the more informative common name for the threat. In a database, the classification entries should be applied as tags that help explain and group the actual specific threats identified. The hierarchical nature of the classification can then be used to aggregate and analyze direct threats at whatever level is most useful. The classification is also useful as a checklist that can be reviewed to make sure a team of practitioners has thought about all possible specific threats across the different categories.

### Limited set of changes needed at higher levels of the classification

There is an inherent tension between not wanting to unduly modify a classification that is used as an international standard and ensuring that the classification corrects omissions and sources of confusion as well as accommodates changing conditions in the world. In reviewing the many citations and uses of previous versions of the classification, we found that the higher levels of the classification did not require substantial modification, indicating that the classification was reasonably robust. In particular, in reviewing the 32,271 direct threats listed in the Miradi database, the biggest problem involved the 2300 factors coded as direct threats but that were actually biophysical factors and the 2298 that were actually contributing factors (see below). The only true direct threats in this analysis that could not be added to existing classifications were 24 entries that corresponded to borders and walls, hence our addition of “4.5 Fencing & walls.” There were also a few specific direct threats that we had not considered before but that we were able to assign to one of the existing level 2 classifications (e.g., livestock rustling, which we assigned to “6.3 Other human disturbances”).

Version 4.0 thus contains no completely new level 1 threats and only one completely new level 2 threat (“4.5 Fencing & walls”). To better meet our comprehensive criterion and to go along with “7.1 Fire & fire management” and “7.2 Dams & water management/use,” we subdivided version 2.0's “7.3 Other ecosystem modifications” into the remaining Aristotelian elements (fire, water, earth, air) of “7.3 Earth & sediment management,” “7.4 Weather & climate management,” and “7.5 Biological system management”. The threat “7.4 Weather & climate management” also became a good place to put all the emerging climate‐geoengineering activities. Finally, we made some changes to “10. Natural disasters” and “11. Climate change” to better reflect the evolution of our understanding of these threats per the discussion below.

### Splitting versus lumping threats

One of the long‐standing tenets of identifying and prioritizing threats is that one should lump threats when they affect the same biodiversity focal values, co‐occur on the land or seascape, result from the same actors, and therefore require similar conservation actions. Conversely, one should split threats if they require different conservation actions. This splitting versus lumping issue spills over into the threat classification itself in some cases that challenge our exclusivity criterion for the ideal classification. Although ultimately it does not matter whether a concept goes into one category or the other, it is helpful to be consistent in how threats are coded.

One example that led to considerable debate among our task force and with our reviewers is where to put various human actions that involve killing animals. Here, the salient information is the motivation for the killing and thus the actions required to mitigate the population‐level impacts of this threat. On one end of the spectrum, hunting or fishing for consumptive purposes clearly belongs in “5.1 Hunting, collecting & controlling terrestrial animals” or “5.4 Fishing, harvesting & controlling aquatic species.” On the other end, it seemed potentially useful to include culling of animals by professional game‐reserve managers in “7.5 Biological system management” alongside other nonlethal control methods, such as translocation or implementation of contraceptives. But in the middle of this spectrum, should killing of animals by local communities in response to human–wildlife conflict be considered hunting (order 5.1) or management (order 7.5)? Similarly, where government wildlife management agencies set hunting quotas to manage wildlife populations in the absence of their natural predators, is this hunting or species management? Even trickier, at which end of this spectrum should killing of animals for persecution reasons (e.g., killing snakes or sharks) be included? All these cases are made even more difficult, of course, because there may be multiple motivations for killing an animal. For example, the meat of culled animals might be sold for human consumption. In the end, we concluded that it is more practical to put all killing of animals under “5. Biological resource use.”

Other examples of where we drew black lines on gray areas of how to split versus lump closely related threats included keeping hydroelectric power dams in “7.2 Dams & water management & use” rather than “3.3 Renewable energy production,” putting biofuel crops in “2.1 Annual & perennial non‐timber crops” rather than “3.3 Renewable energy,” and putting the presence of hikers on trails in “6.1 Recreational activities” rather than (analogous to cars operating on roads) in “4.1 Roads, trails & railroads.” Our recommendations in these cases are captured in the exposition column of the classification. The key is to be consistent in how threats are coded so that analyses based on this classification are standardized.

### Advantages of adding level 3 modifiers

Adding the level 3 modifiers (see Table 4 for an example and Appendix S4 for full details) to the classification provided a more detailed description of different types of direct threats, thus allowing practitioners to fine‐tune the actions needed to counter these threats. Furthermore, this structure allows users to mix and match different types with elements of the various modifiers, creating a powerful and flexible tagging system. For example, one can now distinguish between the bycatch effects of illegal fishing as opposed to the targeted‐species effects of legal subsistence fishing. Likewise, we maintained the level 2 focus on the source of pollutants but have added the pollutant or pollutants of concern as level 3 modifiers. If users apply these modifiers, it should then be possible to track the impact of specific pollutants, such as toxic chemicals or microplastics.

In considering potential level 3 modifiers, we explicitly avoided using components of threat magnitude. Most current threat magnitude assessment approaches involve measuring or rating the scope and severity of a threat's impact on a biodiversity focal value factor (e.g., CMP, 2020; CMP–IUCN, 2007; Master et al., 2012; Wulffraat & Morrison, 2013). In addition, other common components include irreversibility and urgency or temporality. Because these assessments are applied to a direct threat–focal biodiversity value pair, these assessments do not belong in the definition of the threat itself.

### Including pollution and invasive or problematic species as direct threats

One problem with the use of this classification that emerges from our analyses of the Miradi results is that some users are still trying to classify various biophysical factors as direct threats. We deemed 2298 (7%) of the 32,271 factors coded as threats to actually be biophysical stress factors, such as ecosystem degradation or lack of nesting success. To some degree, this confusion can be addressed by providing users with better guidance and examples of the difference between direct threats and stresses, emphasizing that the latter imply a degraded condition of the biodiversity focal value species or ecosystem that results from a direct threat (IUCN, 2012).

This problem also reflects a long‐standing discussion about a number of entries in this classification that seem to blur the distinction between direct threats and biophysical factors that are stressors in the system. For example, where a battery factory is emitting toxic metals into a wetland or human transport has introduced an invasive species onto an island, the battery factory emissions or the transport actions are clearly the human activities that are creating a threat, whereas the toxic metals in the wetland or the invasive species are the resulting stressors. So, it could be confusing to practitioners to see pollution or invasive species listed as potential direct threats. But practically speaking, there are also cases where heavy metals or invasive species in an ecosystem have historical or unknown origins. Even where the source is known, different actions are generally required to stop pollution or invasive species from entering a system as opposed to removing them or managing their impacts. In these cases, it is more practical to identify and rate the pollution or the invasive species as the direct threat that must then be addressed. Indeed, it would probably strike most people as odd to not have pollution or invasive species listed in a classification of potential threats to biodiversity.

We have thus retained these threats in the overall classification. We hope that by grouping them into the new level 0 class “C. Additional sources of stress,” it will become clearer to users why these entries that blur the line between threats and stressors are in the classification. In our guidance, we also encourage users to classify the pollution and invasive species or diseases that result from the various threats in classes “A. Use of lands & waters” and “B. Use/management of species & ecosystems” as their own threats in the appropriate category in class “C. Additional sources of stress” because managing these threats often requires different actions. Ultimately, it is up to users to ensure they are not double‐counting by including the same problem twice in their analyses and prioritizations.

### Including natural disasters as direct threats

An even more extreme example of blurring the line between direct threats and biophysical factors comes from the inclusion of obviously natural phenomena, such as geological events or storms, in the direct threat classification. In a normally functioning ecosystem, these phenomena are natural parts of disturbance regimes that are necessary to maintain long‐term ecosystem function. And yet, previous versions of this threat classification included geological events based on the logic that if a species has a highly reduced population due to other threats and has lost its resilience, then managers must account for the effects of these events. For example, if one is managing the last population of Galápagos pink land iguanas (*Conolophus marthae*) that live in a volcanic caldera that could erupt at any moment or if a large percentage of remaining individuals of a species are in a captive breeding facility that is vulnerable to fire or hurricanes, then clearly one must be concerned about these existential threats to the survival of the species.

This inclusion of these natural phenomena as direct threats obviously muddies the current definition of a direct threat (CMP, 2020). As one reviewer of the classification eloquently stated, if the vulnerability of a reduced population to disturbance justifies including the cause of a disturbance in the direct threats classification, “then any natural process that a species becomes vulnerable to because of a human activity would be considered a threat. This would require that the following also count as threats: natural predators, natural competitors, natural parasites, bad weather, accidents, starvation, seasonality, or anything else that may impact an individual…with the above logic, all these natural factors could be considered threats. But that would make the definition useless; mixing natural processes and human actions would open a can of worms.”

We agree with this reviewer and others who made similar arguments that it is not helpful to include most altered natural processes in the direct threats classification. But we recommend distinguishing endogenous ecosystem processes that managers of remnant populations can generally deal with from large‐scale, unpredictable, and rare exogenous events that could catastrophically affect a biodiversity focal value. Managers must include these latter events in their planning and prioritization work. To this end, we kept this concept as a level 1 order but combined the geological and storm events in it to emphasize that it is their impact on vulnerable populations that is of concern, and we improved the explanation of this concept in the definition and exposition. We also adapted the definition of *direct threat* to better accommodate this concept (see below).

### Capturing threats from climate change

**FIGURE 3:**
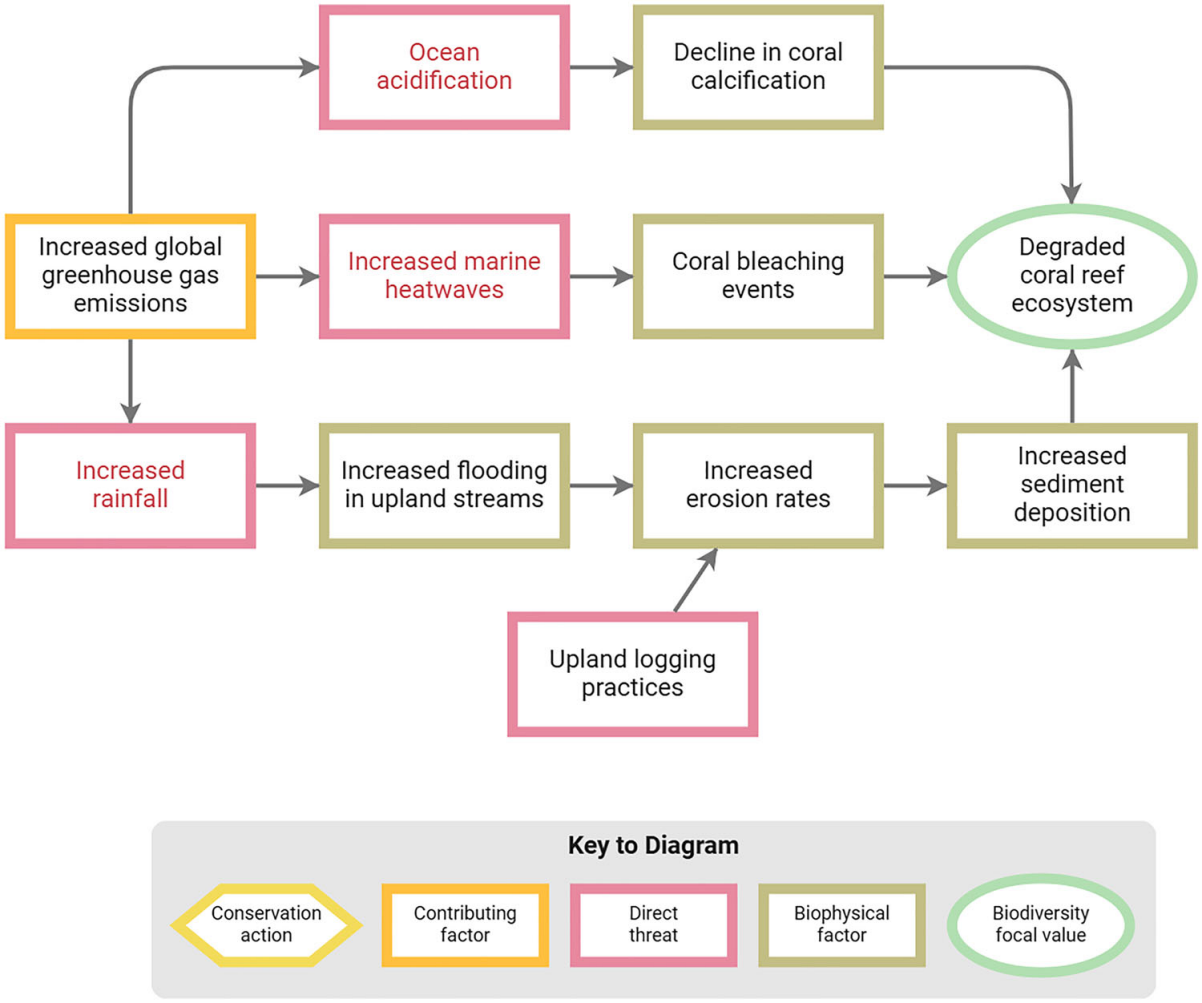
Examples of direct threats from climate change and their associated pathways (red font, distinguishes climate from conventional direct threats).

Capturing climate change in threats analyses has long been tricky. Climate change is a threat in its own right and a force that can amplify and compound the impacts of the other more “conventional” direct threats (Brown et al., 2022). In practice, climate change is usually best described by a pathway of biophysical factors caused by the direct threat of anthropogenic greenhouse gas (GHG) emissions. For example, one such pathway might involve increased storm event frequency and duration (Figure 3), which leads to increased flooding in upland terrestrial streams, which leads to increased erosion rates, which leads to increased sediment deposition, which damages our coral reef ecosystem biodiversity focal value. In general, although practitioners cannot influence the climate factors, they can try to mitigate their effects on ecological systems (e.g., try to manage the effects of sedimentation on a coral reef) or can try to manage conventional direct threats so that the focal ecosystems, species, or both are as resilient as possible (e.g., try to reduce sedimentation caused by upstream logging).

The challenge from a classification point of view is thus what to label the direct threat in this chain. The human action that has ultimately caused this problem is the global GHG emissions, which technically could be coded to our classification as “9.3 Air‐borne pollutants.” But it is not helpful to most conservation practitioners to simply list this nondiscriminating factor as the sole climate‐related direct threat in all conservation situations. Instead, the guidance developed by the CMP (Brown et al., 2022) recommends that practitioners identify and prioritize as “climate direct threats” the most immediate abiotic impacts of climate change moving from left to right along this situation pathway. Thus, in the bottom chain in Figure 3, increased rainfall would be designated as the climate direct threat (pink box with red text). As can be seen in the diagram, there are other climate direct threats in this situation model, such as increased marine heat waves and ocean acidification. Each of these 3 threats is part of a different situation pathway chain that can potentially affect the coral reef biodiversity focal value through different mechanisms with potentially different levels of scope, severity, and timing. To conserve the coral reef focal value, one must ultimately consider all 3 of these pathways. By identifying one direct threat factor in each pathway, each pathway can then be appropriately represented in any subsequent threat‐based prioritization or management efforts.

Readers will note that the biophysical factors in the bottom chain of Figure 3 (increased flooding in upland streams, increased erosion rates, increased sediment deposition) all correspond to level 3 entries in “11 Climate change” in the threats classification and so could theoretically be represented by pink direct threat boxes. But having multiple direct threats in one pathway chain would lead to this chain being overrepresented in any prioritization exercise. We thus recommend that practitioners follow CMP's guidance to only code the ultimate (in this diagram orientation, the left most) factor in each pathway as the climate direct threat. The key is to make sure that all relevant climate pathways have been identified and included in prioritization and management efforts.

### Redefining the term *direct threat*

As discussed above, there is an inherent tension between having a simple and logically consistent definition of *direct threats* and usefully capturing the range of problems that practitioners must prioritize and manage. In the current definition in the Conservation Standards (CMP, 2020), direct threats include “human actions that immediately degrade one or more [biodiversity focal values].” They also include “natural phenomena altered by human activities” such as more frequent and extreme storms due to climate change. But this definition does not fully encompass the range of threats in version 4.0 of the classification. Based on the above discussion, we thus propose to modify this definition: “Direct threats are at the interface between human socioeconomic and natural systems. They include human actions that are the direct cause of ecosystem or species‐population degradation and loss, such as agriculture, transport, natural resource use, and ecosystem management. They also include ultimate stressors in natural systems whose dynamics have been altered by the effects of current or historical human actions, such as invasive or problematic native species, pollution, natural disasters, and climate change.”

By substituting *direct* for *immediately*, we are clarifying that this is primarily a conceptual, as opposed to temporal, relationship. Direct threats include the human actions that are proximately causing the problems in the natural system (factors on the right side of the orange human socioeconomic system box in Figure 1). By including ultimate stressors, we are referring to additional sources of stress that are largely exogenous to focal ecosystems or species populations (factors on the left side of the green natural system box in Figure 1), thus including the impacts of invasive species or tsunamis but excluding endogenous stresses, such as the loss of nesting habitat or of seed dispersers that should be treated as biophysical stress factors and prioritized for management and restoration efforts. This distinction is similar to the one made by IPBES whose online glossary distinguishes between anthropogenic direct drivers that “result from human decisions” and natural direct drivers that “are beyond human control” (IPBES, 2024). We hope that this revised definition, which is also reflected in our new level 0 classes, will help practitioners better understand and use the concept of direct threats in their conservation work.

### Toward the next iteration of this classification

Overall, we, the task force members, believes that version 4.0 of the classification substantially improves on previous versions. As such, we hope it will be adopted and used as appropriate. However, it can still be improved, including, in particular, further development of the level 3 and 4 entries. We look forward to continuing to receive users’ and critics’ suggestions and feedback that can be used to inform future revisions of this work.

## Supporting information



Appendix S1. Numbers of citations of Version 1.0 of this classification (Salafsky et al. 2008) over time per Google Scholar as of November 2023. Total citations = 1025.

Appendix S2. Proportion of threats classified incorrectly in each Level 1 Threat Order. The table is sorted from highest to lowest proportion of discrepancies.

Appendix S3. Classification Levels 0‐2 + examples, definitions, expositions, and mapping to v 2.0

Appendix S4. Classification Levels 0‐2 plus Level 3 Types & Modifiers and Level 4 Types
