# Supplementary figures and images for: Classification of direct threats to the conservation of ecosystems and species 4.0
Source: Conserv Biol. 2024 Dec 31;39(3):e14434. doi: 10.1111/cobi.14434 (PMC12124163; doi:10.1111/cobi.14434)

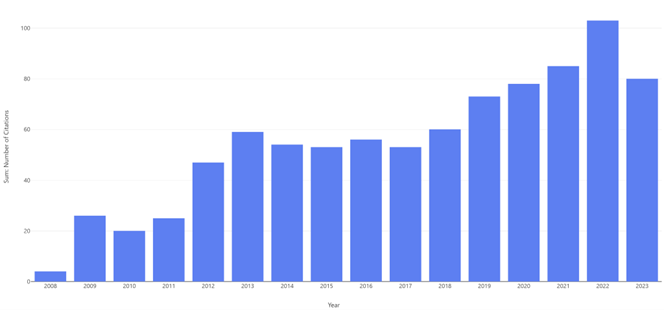

Supplement: Supplementary file 1 — Appendix S1. Numbers of citations of Version 1.0 of this classification (Salafsky et al. 2008) over time per Google Scholar as of November 2023. Total citations = 1025. [file COBI-39-e14434-s004.png]
