# Supplementary material for: Classification of direct threats to the conservation of ecosystems and species 4.0
Source: Conserv Biol. 2024 Dec 31;39(3):e14434. doi: 10.1111/cobi.14434 (PMC12124163; doi:10.1111/cobi.14434)
Supplement: Supplementary file 2 — Appendix S2. Proportion of threats classified incorrectly in each Level 1 Threat Order. The table is sorted from highest to lowest proportion of discrepancies. [file COBI-39-e14434-s002.docx]

***Appendix S2.*** *Proportion of threats classified incorrectly in each Level 1 Threat Order. The table is sorted from highest to lowest proportion of discrepancies.*

| **Level 1 Threat Order** | **Coded Incorrectly (%)** | | | **Coded Correctly (%)** | | |
| --- | --- | --- | --- | --- | --- | --- |
|  | **Discrepancy outside of Level 1 class** | **Discrepancy within Level 1** | **TOTAL** | **Consistent to Level 1** | **Consistent to Level 2** | **TOTAL** |
| 11. Climate Change | 3.3 | 27.5 | **30.8** | 16.2 | 53.0 | **69.2** |
| 7. Natural System Modifications | 17.6 | 10.4 | **28.0** | 17.1 | 54.8 | **72.0** |
| 8. Invasive & Problematic Species, Pathogens & Genes | 1.4 | 19.8 | **21.2** | 19.5 | 59.3 | **78.9** |
| 6. Human Intrusions & Disturbance | 8.4 | 1.7 | **10.1** | 19.1 | 70.8 | **89.9** |
| 9. Pollution | 6.2 | 1.2 | **7.4** | 13.6 | 79.0 | **92.6** |
| 4. Transportation & Service Corridors | 6.1 | 1.0 | **7.1** | 19.0 | 74.0 | **92.9** |
| 2. Agriculture & Aquaculture | 6.1 | 0.6 | **6.6** | 17.2 | 76.2 | **93.4** |
| 5. Biological Resource Use | 2.4 | 2.6 | **5.0** | 18.3 | 76.7 | **95.0** |
| 1. Residential & Commercial Development | 2.6 | 1.6 | **4.2** | 23.9 | 71.9 | **95.8** |
| 3. Energy Production & Mining | 2.7 | 0.4 | **3.1** | 23.2 | 73.8 | **96.9** |
| 10. Geological Events | 0.0 | 2.1 | **2.1** | 0.0 | 97.9 | **97.9** |

*Of the 16,169 factors coded by their users to Version 1.0 of the classification, 16,145 were assessed for whether the users had coded them correctly to either the Level 1 or Level 2 threat level and corrected them where necessary. The remaining 24 were marked as unknown threats, as the threat name and description were indecipherable. The majority of factors were coded correctly to Level 2. Orders that were commonly misclassified reflected some areas of the Version 1.0 classification that required better explanation, improved titles, more examples and/or potential adaptation. Some of these issues had already been addressed in Version 2.0, however, a few remaining discrepancies proved useful for prompting additional adaptations made in Version 4.0.*
