# Supplementary material for: Classification of direct threats to the conservation of ecosystems and species 4.0
Source: Conserv Biol. 2024 Dec 31;39(3):e14434. doi: 10.1111/cobi.14434 (PMC12124163; doi:10.1111/cobi.14434)
Supplement: Supplementary file 3 — Appendix S3. Classification Levels 0‐2 + examples, definitions, expositions, and mapping to v 2.0 [file COBI-39-e14434-s001.pdf]

Table 3a. Classification Levels 0-2 + examples, definitions, expositions, and mapping to v 2.0

IUCN - CMP Direct Threats Classification v 4.0<br>0 Threat Class<br>L1 L2 *(examples, not comprehensive)*	Definition	Exposition	Mapping to CMP v 2.0
**A. Use of Lands & Waters**	Human uses of land and water areas that have a substantial spatial footprint. Includes effects from their construction (e.g. ecosystem conversion), ongoing use, and abandonment.	This class includes both the ecosystem conversion / degradation effects of the expansion and ongoing presence of these activities, but does not include their associated pollution effects. The threats in *1. Residential, Commercial & Recreation Areas* and *2. Agriculture & Aquaculture* are generally tied to a defined and relatively compact area, which distinguishes them from those in *4. Transportation, Service & Security Corridors* which have a long narrow footprint, and *6. Human Intrusions & Disturbances* which do not have an explicit footprint. Standard land-cover classifications can often be used to assess the stresses delivered by these direct threats.	We are adding this "0 Level" to the classification to help users understand the different threat types.
**1. Residential, Commercial & Recreation Areas**	Human settlements, industrial areas, and other non-agricultural land uses with a substantial footprint.	There is potential overlap between the threats in these categories. For example, should a tourist hotel complex in a city be part of 1.1 or 1.3? In general, most activities inside a defined municipal area should be in 1.1 wheras 1.2 and 1.3 are more for stand-alone developments in otherwise natural spaces.	Edits to the name of the threat.
**1.1 Residential Areas**<br>*urban areas, suburbs, villages, rural housing, vacation homes, shopping areas, offices, schools, hospitals, houses of worship*	Cities, towns, and settlements including non-housing development typically integrated with housing.	This category dovetails somewhat arbitrarily with *1.2 Commercial & Industrial Areas.* As a general rule, however, if people live in or directly around the footprint of the area in question, it should fall into this category. Tourism facilities within a municipal area should generally go here and not in *1.3 Recreation & Tourism Areas*.	Edits to the name of the category to empahsize residential nature.
**1.2 Commercial & Industrial Areas**<br>*stand-alone office parks, stand-alone shopping centers, manufacturing plants, military bases, coal or nuclear power plants, sewage treatment plants, landfills, train yards, ports, airports, land reclamation for industrial development*	Factories and other commercial centers.	Ports and airports fall into this category, whereas shipping lanes and flight paths fall under *4. Transportation, Service & Security Corridors.* Hydropower dams are NOT included here, but are in *7.2 Dams & Water Management / Use.*	
**1.3 Recreation & Tourism Areas**<br>*visitor facilities in parks, campgrounds, football or other sports fields, ski areas, golf courses, beach facilities, fishing piers, marinas, cultural tourist attractions, pilgrimage facilities, cemeteries*	Tourism and recreation sites with a substantial spatial footprint that are not fully integrated into municipal areas.	This category focuses on the spatial footprint of recreation areas and facilities while *6.1 Recreational Activities* focuses on the disturbance effects posed by recreational activities. There is a fine line between residential areas and tourism/resort areas; per discussion above, if the tourism area is within municipal boundaries, it probably belongs in *1.1 Residential Areas*. Trails and other linear tourism features belong in *4.1. Roads, Trails & Railroads*.	Edits to the name of the category and definition.
**2. Agriculture & Aquaculture**	Farming and ranching including agricultural expansion, intensification, or practices with a spatial footprint; includes tree plantations, mariculture, and aquaculture.	This order focuses on the footprint and operations of these activities. Agricultural and aquacultural pollution threats (e.g. drift of herbicides or run-off of fertilizers) should be included in the appropriate category under *9. Pollution*.	Minor edits to the definition.
**2.1 Annual & Perennial Non-Timber Crops**<br>*farms, household swidden plots, oil palm plantations, orchards, vineyards, mixed agroforestry systems, biofuel crops, haying practices that disturb nesting birds*	Crops planted for food, fodder, fiber, fuel, or other uses.	"Shifting cultivation" refers to systems in which land is temporarily farmed and then abandoned for a period of time, not crop rotation or occasional fallow periods on annual crop lands. Crops grown in on-farm greenhouses belong here; those grown in urban greenhouses, vertical farm facilities, or in indoor 'factories' (e.g. marijuana) should be included in *1.2 Commercial & Industrial Areas*.	
**2.2 Wood & Pulp Plantations**<br>*teak, eucalyptus or acacia plantations, pulp or fiber plantations, firewood lots, christmas tree farms*	Stands of trees planted for timber or fiber outside of natural forests, often with non-native species.	If it is one or a couple timber species that are planted on a rotation cycle, it belongs here. If it is multiple species or enrichment plantings in a quasi-natural system, it belongs in *5.3 Logging, Harvesting & Controlling Trees.*	
**2.3 Terrestrial Animal Farming, Ranching & Herding**<br>*cattle feed lots, dairy farms, cattle ranching, chicken farms, goat, camel, or yak herding, game farms, bee keeping*	Domestic terrestrial animals raised in one location on farmed or non-local resources (farming); also domestic or semidomesticated animals allowed to roam in semi-natural areas (ranching) or the wild and supported by natural habitats (herding).	In farming, animals are kept in tight captivity; in ranching they are allowed to roam in larger more natural areas, and in herding they are using wild habitats. If a few animals are mixed in a subsistence cropping system, it belongs in *2.1 Annual & Perennial Non-Timber Crops.* Foraging for wild resources for stall-fed animals falls under *5.2 Gathering, Collecting & Controlling Terrestrial Plants & Fungi.* Growing crops for animal consumption falls under *2.1 Annual & Perennial Non-Timber Crops.* Producing meat from animal cells in factories belongs in *1.2 Commercial & Industrial Areas.*	Edits to the name of the category and definition.
**2.4 Marine & Freshwater Aquaculture**<br>*fish ponds on farms, shrimp production in artificial ponds, salmon production in pens in natural water, hatchery salmon, seeded shellfish beds, artificial algal beds*	Aquatic species raised for harvest in artificial water bodies (analogous to terrestrial 'farming'), enclosures in natural waters (analogous to 'ranching'), or unenclosed natural waters (analogous to 'herding').	It may seem strange to talk about 'farming, ranching, and herding" in an aquatic environment, but from a conservationist's point of view, the effects of each are generaly analagous to their terrestrial counterparts. For convenience, we are including raising of aquatic plants, alage, and other non-animal species in this category. Note that producing aquatic organisms for commercial or recreational fishing belongs here and not in *5.4 Fishing, Harvesting & Controlling Aquatic Species*. Producing them for conservation or restoration purposes belongs in *7.5 Biological System Management*. Problems caused by escaped invasive or problematic animals, interbreeding with native species, disease transmission, and pollution from aquaculture should be be coded in the appropriate categories in *C. Additional Sources of Stress*.	Edits to the definition.
**3. Energy Production & Mining**	Extraction of non-biological resources, often widely dispersed across the land /sea scape.	This order contains activities that are generally more widely dispersed than the industrial sites in *1.2 Commercial & Industrial Areas*. While they technically 'produce' energy, power plants and oil refineries are compact industrial sites that belong in *1.2 Commercial & Industrial Areas.* Various forms of water use (for example, dams for hydro power) could conceivably be put in this order, but seem more related to other threats that involve alterations to hydrologic regimes. As a result, they should go in *7.2 Dams & Water Management / Use.*	Minor edits to the definition.
**3.1 Oil & Gas Exploration & Extraction**<br>*oil wells, hydraulic fracturing, deep sea natural gas drilling*	Exploring for and extracting petroleum and other liquid hydrocarbons.	Oil refineries, LPG gas ports, and other activities with a compact footprint belong in *1.2 Commercial & Industrial Areas*. Oil and gas pipelines go into *4.2 Utility & Service Lines.* Oil spills that occur at the drill site or from oil tankers or pipelines should go in *9.1 Water-Borne & Other Effluent Pollution.*	Edits to the name of the category and definition.

**3.2 Mining & Quarrying**<br>*coal mines, alluvial gold panning, gold mines, rock quarries, sand or salt mining, coral mining, deep sea nodules, guano harvesting*	Exploring for, developing and producing minerals and rocks.	It is a judgement call whether deforestation caused by strip mining should be in this category or in *5.3 Logging, Harvesting & Controlling Trees* - it depends on whether the primary motivation for the deforestation is access to the trees or to the minerals. Sediment or toxic chemical runoff from mining should be placed in *9.1 Water-Borne & Other Effluent Pollution* if it is the major threat from a mining operation.	
**3.3 Renewable Energy**<br>*geothermal power production, solar farms, wind farms (including birds or bats flying into windmills), tidal farms*	Exploring, developing and producing renewable energy.	Arguably renewable energy production with a compact footprint (e.g. solar concentrators) could be put in *1.2 Commercial & Industrial Areas* but we propose they belong here to keep it with other forms of renewable energy. Hydropower should be put in *7.2 Dams & Water Management / Use.* Growing biofuels should be in *2.1 Annual or Perennial Non-Timber Crops* or *2.2. Wood & Pulp Plantations*. Wood pellet production from natural forests should be in *5.3 Logging, Harvesting & Controlling Trees.*	Edits to the name of the category.
**4. Transportation, Service & Security Corridors**	Linear infrastructure such as long, narrow service or transport corridors including the effects associated with their use (e.g. mortality from vehicle collisions, restriction of species movement).	This order focuses on corridors outside of human settlements and industrial developments. These corridors create specific stresses to biodiversity including especially loss and fragmentation of habitats and direct killing of wildlife and lead to other threats including the spread of farms, invasive species, and poachers (but note that we are not saying those knock on threats should be coded here). Permeabilty refers to the degree to which the feature creates a barrier to movement and thus fragments the ecosystem and/or species populations of interest.	Edits to the name of the threat and the definition.
**4.1 Roads, Trails & Railroads**<br>*highways, secondary roads, logging roads, hiking or biking trails, bridges & causeways, vehicle collisions with wildlife, public transport systems, railroads*	Transport on roadways and dedicated tracks.	If the main threat is pollution from roads, it belongs in the appropriate category in *9. Pollution*. Off-road vehicles are treated in the appropriate category in *6. Human Intrusions & Disturbance.* If there are small roads associated with a major utility line, they belong in *4.2. Utility & Service Lines.* Technically, a compact trail network on a site could go in *1.3 Recreation & Tourism Areas* and hiking or biking off of a trail belongs in *6.1 Recreational Activities*. That said, the vast majority of linear trails should now go here to the extent that they are considered a threat, although the disturbance effects of trail users still goes in *6.1 Recreational Activities*.	Minor changes to definition.
**4.2 Utility & Service Lines**<br>*electrical & phone wires, aqueducts, oil & gas pipelines, electrocution of wildlife on powerlines*	Transport of energy & resources.	Cell phone and other communication towers belong here. If there are small utility lines using a road right of way, they belong in *4.1 Roads, Trails & Railroads.* Oil spills from pipelines should go in *9.1 Water-Borne & Other Effluent Pollution.* Herbicides flowing or drifting from right of way treatment belongs in the appropriate category under *9. Pollution*.	
**4.3 Shipping Lanes**<br>*maintenance of shipping channels, canals, shipping lanes, ships running into whales, wakes from cargo ships*	Transport on and in freshwater and ocean waterways.	This category includes all larger ships moving on water (i.e. both inside and outside of designated 'lanes'). This category includes dredging and other activities that maintain shipping lanes. Dredging for other purposes belongs in *7.3 Earth & Sediment Management*. Anchor damage from dive boats belongs in *6.1 Recreational Activities*.	
**4.4 Atmospheric & Space Activities**<br>*flight paths, jets impacting birds, commercial or military drones, tethered balloons*	Air and space transport and other activities.	Airports fall into *1.2 Commercial & Industrial Areas.*	Edits to the name of the category and definition.
**4.5 Fencing & Walls**<br>*border walls, fences around farm fields, fences or noise barriers along roads, protected area fencing, disease control fencing*	Barriers to movement.	Small-scale fencing can be incorporated as part of houses or other features, but larger-scale perimeter fencing around houses, factories or farms or linear fencing along roads now should go here because those landscape features could be managed with or without fencing. Even fencing that is designed to protect some wildlife (eg around a game park) may pose a threat to other species.	This is a new category. Fencing and walls may have been mixed in many other categories.
**B. Use / Management of Species & Ecosystems**	Human uses of biotic resources and disturbance from human presence or managment actions in natural systems.	Human actions in this group generally do not 'intend' to convert the ecosystem athough the most destructive forms of logging, fishing or dam building can have this result. These uses typically do not have a large spatial footprint excepting again the most intense forms of resource extraction or ecosystem management.	We are adding this "0 Level" to the classification to help users understand the different threat types.
**5. Biological Resource Use & Control**	Consumptive use of "wild" biological resources including deliberate and unintentional harvesting [illegible]	Consumptive use means that the resource is removed from the system or destroyed; multiple people cannot use the same resource as they could under *6. Human Intrusions & Disturbance.* Threats in this class can affect targeted species (harvest [illegible]	Edits to the name of the threat and definition.
**5.1 Hunting, Collecting & Controlling Terrestrial Animals**<br>*subsistence hunting, collection of feathers or skins used in traditional ceremonies, commercial wild meat hunting, trophy hunting, fur trapping, insect collecting, pet trade, honey or egg collection, persecution of snakes, culling of deer, killing of crop-raiding animals*	Hunting or trapping terrestrial wild animals for commercial, recreation, subsistence, research, or cultural purposes, or killing them for persecution or control reasons; includes non-lethal animal product harvesting and accidental mortality/bycatch.	This category focuses on animals that primarily live in a terrestrial environment. There are obviously some species that live on the terrestrial/aquatic boundary. Hunting otters, beavers, amphibians, polar bears, penguins, waterfowl, and sea birds should (somewhat arbitrarily) go here. Hunting seals, whales and other marine mammals, and freshwater and marine turtles go in *5.4 Fishing, Harvesting & Controlling Aquatic Species.* Yes, most people "gather" honey, eggs, insects or other slow moving targets, rather than "hunt" them. But it seems cleaner to keep all animal products as being hunted. Persecution of animals also belongs here, as does killing of animals to manage their population numbers, impacts on the ecosystem or other species, or their impacts on crops, livestock, or human populations (i.e. because of human-wildlife conflict).	Edits to the name of the category and definition.
**5.2 Gathering, Harvesting & Controlling Terrestrial Plants & Fungi**<br>*gathering wild fruit, mushrooms, orchids, rattan, lichen, or herbs for traditional medicine, collecting forage for stall fed animals, non-woody biomass harvesting, rubber or maple syrup tapping, control of host plants to combat timber diseases*	Harvesting plants, fungi, and other non-timber/non-animal products for commercial, recreation, subsistence, research, or cultural purposes, or persecution or control reasons; includes non-lethal product harvesting and accidental mortality/bycatch.	This category focuses on plants, fungi, and all other non-animal terrestrial species (e.g. chromists) except timber trees which are treated in *5.3 Logging, Harvesting & Controlling Trees*. Includes lethal as well as non-lethal collection methods such as sap tapping. Also includes control of plant and other species to manage their impacts on the ecosystem, other species, or agricultural or forestry operations.	Edits to the name of the category and definition.
**5.3 Logging, Harvesting & Controlling Trees**<br>*clear cutting of hardwoods, selective commercial logging of ironwood, pulp operations, woody biomass collection, fuel wood collection, charcoal production, coppicing, tree thinning*	Harvesting trees and other woody vegetation for timber, fiber, or fuel, including site preparation and other forestry management practices; includes non-lethal coppicing and accidental mortality/bycatch.	Felling trees to clear agricultural land goes in the appropriate category in *2. Agriculture & Aquaculture.* If it is a few timber species that are planted on a rotation cycle, it belongs in *2.2 Wood & Pulp Plantations.* If it is multiple species or enrichment plantings in a quasi-natural system, it belongs here. Also includes logging to manage or control forests, unless it it is primarily for fire management which belongs in *7.1 Fire & Fire Management.*	Edits to the name of the category and definition.

**5.4 Fishing, Harvesting & Controlling Aquatic Species**<br>*net fishing, hook and line fishing, trawling, blast fishing, spear fishing, shellfish harvesting, whaling, seal hunting, turtle egg collection, live coral collection, aquarium fish collection, seaweed collection, persecution of sharks, control of seals*	Harvesting aquatic wild animals or plants for commercial, recreation, subsistence, research, or cultural purposes, or persecution or control reasons; includes non-lethal product harvesting and accidental mortality/bycatch.	This category focuses on all kinds of species that are primarily found in an aquatic environment. There are obviously some species that live on the terrestrial/aquatic boundary. Hunting otters, beavers, amphibians, polar bears, penguins, waterfowl, and sea birds should (somewhat arbitrarily) go in *5.1 Hunting & Collecting Terrestrial Animals.* Hunting seals, whales and other marine mammals, and freshwater and marine turtles go here.	Edits to the name of the category and definition.
**6. Human Intrusions & Disturbances**	Human activities that alter, disturb, and destroy ecosystems and species associated with non-consumptive uses of biological areas and resources.	Non-consumptive use means that the resource is not removed - multiple people can use the same resource (for example, birdwatching). These threats typically do not permanently destroy ecosystems except in extremely severe manifestations. Pollution from these activities belongs in the appropriate category in *9. Pollution.*	Edits to the name of the threat and definition.
**6.1 Recreational Activities**<br>*hikers, mountain bikes, horse riding, off-road vehicles, motorboats, jet-skis, snowmobiles, ultralight planes, temporary campsites, caving, rock-climbing, dive boats, whale watching boats, birdwatchers, pets in recreational areas*	People spending time in natural areas or traveling in vehicles outside of established transport corridors, usually for recreational reasons.	This category does not include direct or indirect effects of consumptive use of biodiversity - for example hunting and logging or disturbance impacts from loggers or hunters would be in the appropriate category in *5. Biological Resource Use & Control.* Non-recreational vehicles, boats, and other human movement in established transport corridors go in *4. Transportation, Service & Security Corridors,* except the presence of trail users both on and off trails belongs here. The development of permanent recreational or tourist facilities (such as hotels and resorts) should be included under *1.3 Tourism & Recreation Areas* rather than here.	Minor edits to the definition.
**6.2 Conflict, Civil Unrest & Security Activities**<br>*armed conflict, riots, military training exercises, border patrols, peace keeping activities, guerilla camps, abandoned land mines, defoliation, munitions testing*	Actions in natural areas by formal or paramilitary forces without a permanent footprint.	This category focuses on military or security activities that have a large impact on natural habitats, but are not permanently restricted to a single area. Permanent military bases should go under *1.2 Commercial & Industrial Areas.* Other military activities might best be assigned to other categories. For example, hunting of specific animals by soldiers living off the land fits under *5.1 Hunting, Collecting & Controlling Terrestrial Animals.* Permanent security fences fit under *4.5 Fencing & Walls.*	Edits to the name of the category and definition.
**6.3 Other Human Disturbances**<br>*drug smuggling or human migration through natural areas, livestock rustling, household water collection, non-recreational bathing in rivers, festivals, pilgrimages, species research, archeological research, vandalism*	People spending time in or traveling in natural environments for reasons other than recreation or conflict/security activities	This category is used when human presence is the main stress of the activity. It is not used if the human presence is part of a different threat like hunting or logging.	Edits to the name of the category and definition.
**7. Natural System Management & Modifications**	Human actions that modify ecosystem structures, composition, or regimes, generally to deliberately improve human welfare or benefit certain species. This category includes both construction of permanent or long-term structures and their operations as well as more transitory management practices.	This order deals primarily with human caused changes to natural ecosystem processes such as fire, hydrology, and sedimentation, rather than land use. Thus it does not include threats relating to infrastructure (*1. Residential & Commercial Development* and *4. Transportation, Service & Security Corridors*) or agriculture (which should be under *2. Agriculture & Aquaculture*). It also includes the removal of management actions on which ecosystems and species now depend.	Edits to the name of the threat.
**7.1 Fire & Fire Management**<br>*fire suppression to protect homes, inappropriate fire management, building of fire breaks, escaped agricultural fires, arson, campfires, fires for hunting*	Management actions that either suppress or increase fire frequency and/or intensity.	This category focuses on the human activities that lead to either not enough fire or too much fire in the ecosystem in question. If fire escapes from established agricultural lands, it belongs here, if fire is used to clear new agricultural lands, it belongs in the appropriate category in *2. Agriculture & Aquaculture.* It also includes damaging "natural" fires in systems that have lost their natural resilience. While suppression of traditional or cultural fire practices could be placed in *7.6 Removing / Reducing Human Management*, we suggest keeping it here to have all the fire threats in one category.	Edits to the name of the category and definition.
**7.2 Dams & Water Management / Use**<br>*dam construction, dam operations, levees and dikes, channelization, highway culverts, adding drains to wetlands for mosquito control, removal of natural beaver dams, encouraging beaver dams, water catchment areas, snow fences, dew harvesting, surface water withdrawals, groundwater pumping, increased humidity from human water use, water desalination, artificial lakes, birds drowning in artificial reservoirs, water treatment plants, adding lime to acid lakes*	Management actions that modify water levels, flows, and chemistry.	This category focuses on the human activities that alter or fragment aquatic habitats, alter water flows and chemistry, and/or lead to either not enough water or too much water in the ecosystem in question. Hydropower dams could have also been in *3.3 Renewable Energy*, but are placed here to keep all dams together. Dredging and channelization for shipping belongs in *4.3 Shipping Lanes;* other dredging activities belong in *7.3 Earth & Sediment Management.*	Minor edits to the definition.
**7.3 Earth & Sediment Management**<br>*dune stablization, sediment fencing, shoreline armoring, beach groins, soil pollution remediation, mine reclamation, land reclamation, dredging (except for shipping lanes)*	Management actions that modify the geophysical environment and/or change sediment regimes.	Excess sediment itself belongs in the appropriate category in *9. Pollution*. Reduced sediment caused by dams should be attibuted to 7.2 Dams & Water Management / Use. It's a bit arbitrary whether shoreline modifications belong here or in *7.2 Dams & Water Management / Use*. As a general rule, if the modifications are aimed at controlling the water, they belong in 7.2 whereas if they are controlling the sediment or land, they belong here. Although sediment is carried by both wind and water, all sediment management is put here.	This is a new category split from the old 7.3.
**7.4 Weather & Climate Management**	Management actions that modify atmospheric [illegible]	This is a rapidly expanding category that will likely encompass many new actions as we seek to mitigate and adapt to [illegible]	This is a new category split from [illegible]

cloud seeding, frost prevention, iron 'fertilization' in the ocean, releasing reflective particles in the atmosphere, Lagrange point shades, carbon capture (except for infrastructure)	structure and processes.	climate change. Carbon capture plants with a limited footprint probably belong in *1.2 Commercial & Industrial Areas* along wih other factories and power plants.	the old 7.3.
**7.5 Biological System Management**<br>*mowing grass, using cattle to mimic natural grazing processs, removal of snags from streams, gating caves, artificial reef creation, assisted migration, bird feeders, electric barriers to stop invasive fish passage*	Management actions that modify biotic systems including conservation actions that may have detrimental impacts on other non-targeted species or ecosystems.	This category has potentially tricky overlaps with several other categories. Given that it is difficult to distinguish between killing elephants that are raiding crops or hunting seals for predator control purposes from hunting of these species, we are proposing to put all killing or taking of animal species in *5.1 Hunting, Collecting & Controlling Terrestrial Animals* or *5.4. Fishing, Harvesting & Controlling Aquatic Species*. Likewise, thinning of trees should generally be considered as silvicultural treatments in *5.3 Logging, Harvesting & Controlling Trees* or in *7.1 Fire & Fire Management* depending on the purpose of the action. Finally, we propose putting stocking fish & game species in either *8.1 Invasive Non-Native / Alien Species* or *8.2 Problematic Native Species*. Note that this can take place at different scales - for example, fixing cracks in houses could eliminate key gecko habitat.	This is a new category split from the old 7.3.
**7.6 Removing / Reducing Human Management**<br>*lack of mowing of meadows, cessation of grazing, removal of livestock and hence loss of dung, stopping predator control, reduction or loss of Indigenous management of key ecosystems, ceasing supplemental feeding of condors*	Absence or reduction of current or historical management regimes important for maintaining desired key ecological attributes of ecosystems or species. Includes regimes historically maintained by protected area staff, farmers and ranchers, indigenous peoples, private landowners, or any other resource manager.	Although there is a great deal of debate about the role and appropriateness of human actions in shaping 'natural' systems, it is clear that many ecosystems and species depend on human maintenance to mimic natural conditions and maintain key attributes. This threat captures the loss of these direct maintenance regimes. Caution should be used in applying this category – it is not meant as a catch-all for a lack of conservation action at a site, but rather refers to instances where a historical management action has been reduced or eliminated due to, for example, funding, institutional constraints, or Indigenous actors being disempowered. This category does not include indirect maintenance actions, such as lack of outreach or lack of adequate policy. As noted above, suppression of tradiitonal fire practices should be coded in *7.1 Fire & Fire Management* and suppression of traditional water management practices should be be coded in *7.2 Dams & Water Management / Use*.	Numbering has changed from 7.4 to 7.6. Edits to the name of the category and the definition.
**C. Additional Sources of Stress**	Stressors in natural systems that have been altered by the effects of current or historical human actions.	Many of the entries in this class are the result of other direct threats. For example, agricultural practices or commercial shipping could lead to invasive species, pollution from toxic chemicals, or the release of greenhouse gasses that drive climate change. But there are many situations in which invasive species, pollution, or climate change impacts might be a problem in a project area, but it is not clear what the source of these threats are. It is also often helpful to consider the entries in this category separately in any threat prioritization exercise since they often can be addressed with separate conservation actions. In other cases (e.g. problematic native species, geological or storm events), these entries represent 'natural' sources of stress that nonetheless need to be considered in highly altered natural systems.	We are adding this "0 Level" to the classification to help users understand the different threat types.
**8. Invasive / Other Problematic Species, Genes & Pathogens**	Threats from non-native and native plants, animals, pathogens/microbes, or genetic materials that have or are predicted to have harmful effects on biodiversity following their introduction, spread and/or increase in abundance or virulence.	We restrict the use of "invasive species" to non-native species to keep things simple for policy makers. "Problematic native species" are native species that have become superabundant or otherwise cause problems due to human alterations of the ecosystem."Pathogens" are generally microorganisms that directly cause disease in individual organisms. Microorganisms that are NOT pathogens belong in either *8.1 Invasive Non-Native / Alien Species* or *8.2 Problematic Native Species*.	Edits to the name of the threat.
**8.1 Invasive Non-Native / Alien Species**<br>*rats on islands, feral horses, nonferal household pets, feral household pets, zebra mussels, bamboo, introduction of species for biocontrol, stocking exotic fish, ballast water discharge*	Harmful plants, animals, and other species not originally found within the ecosystem(s) in question and directly or indirectly introduced and spread into it by human activities.	We are defining non-native/alien/exotic species as those spread intentionally, accidentally, or faciliated by humans, excluding pathogens. Level 4 can be used to identify the specific taxa of concern; some species may only be identifiable to a higher order taxonomic group. In light of climate change driven shifts of ecosystems and species, it may require a judgement call to determine when a species is "invading" an ecosystem versus merely "naturally migrating."	Edits to the name of the category.
**8.2 Problematic Native Species**<br>*overabundant native deer, algal blooms, insect outbreaks*	Harmful plants, animals, and other species that are originally found within the ecosystem(s) in question, but have become "out-of-balance" or "released" directly or indirectly due to human activities.	This category also excludes pathogens. It is a bit of a judgement call as to when a species becomes "problematic" (aka outside its natural range of variation), especially in the face of climate driven shifts. In many cases, the root cause of the problematic species may be other threats (e.g. hunting that removes predators that then causes their prey species to become hyper abundant). In these cases, it is also a judgement call as to whether the threat should be the hunting or the problematic species. Level 4 can be used to identify the specific taxa of concern.	Edits to the name of the category.
**8.3 Introduced Genetic Material**<br>*pesticide resistant crops, hatchery salmon breeding with wild fish, domestic cats breeding with wild cats, restoration projects using non-local seed stock, genetically modified insects for biocontrol, genetically modified trees, genetically modified salmon or shellfish*	Human caused introduction of natural or synthetic genes into species in natural ecosystems.	As a general rule, introduction of entire organisms (obviously incuding their genetic material) belongs in 8.1 and 8.2. However, if the primary issue is hybidization with native species (e.g. hatchery salmon), then species introduction belongs in this category. This category will likely dramatically expand with the advent of new genetic technology.	Edits to the definition.
**8.4 Pathogens**<br>*plague affecting rodents, chronic wasting disease affecting cervids, Dutch elm disease or chestnut blight, Chytrid fungus affecting amphibians, seastar wasting disease*	Harmful native and non-native agents that cause disease or illness to a host species, including bacteria, viruses, prions, fungi, and other microorganisms.	We are distinguishing between microorganism pathogens that directly cause disease in individual organisms as opposed to invasive or problematic macroorganism species which impact ecosystems. Pathogens that cause disease levels that are within the "natural or acceptable range of variation" for a species population are not a threat. In cases in which invasive or problematic native species serve as vectors for pathogen transmission, users probably will want to focus on the organisim that can be managed as the direct threat. Level 4 can be used to identify the specific taxa of concern.	Edits to the name of the category and definition.
**9. Pollution**	Introduction of exotic and/or excess materials or energy from point and nonpoint sources.	This order deals with exotic or excess materials introduced to the environment, which often have a different spatial area of impact than their source human activity. There is obviously a fine distinction when the pollution comes from another threat - for example, should an oil spill from a pipeline be classified as *4.2 Utility & Service Lines* or *9.1 Water-Borne & Other Effluent Pollution*? You will have to exercise some judgement as to which represents the direct threat in your situation. In some cases, the source of the pollution may be either unknown or from a historical source (e.g., heavy metals buried in sediments). In these cases, you may have to make an educated guess as to which category to assign the pollutant. The new Level 3 classifications help articulate both the source of the pollution as well as the type of pollutant.	Edits to the definition.

**9.1 Water-Borne & Other Effluent Pollution**<br>*discharge from municipal waste treatment plants, leaking septic systems, untreated sewage, outhouses, fertilizers and pesticides from lawns and golf-courses, toxic chemicals from factories, illegal dumping of chemicals, nutrient loading from fertilizer run-off, herbicide run-off, manure from feedlots, excess nutrients from aquaculture, oil spills from pipelines, leaching from mine tailings, arsenic from gold mining, leakage from fuel tanks, oil or sediment from roads, road salt, erosion from logging operations, toxic chemicals in dredged river sediments*	Water-borne and other liquid pollutants stemming from various human activities; includes both the effects of these pollutants on the sites where they are generated/applied and where they end up in the environment.	This category includes sewage and industrial waste as well as the application of toxic chemicals (e.g. insecticides and herbicides) in farming and forestry operations. However, if these chemicals are drifting in the air, they should be coded as part of *9.3 Air-Borne Pollutnts*. Although acid rain is technically liquid borne, it should be coded as part of *9.3 Air-Borne Pollutants*. Note that the Level 3 Source Type deliberately includes the various threats in Classes A & B following the logic that we want to separate out pollution from its source human activities.	This category merges the old *9.1 Household Sewage & Urban Waste Water*, *9.2 Industrial & Military Effluents*, and *9.3 Agricultural & Forestry Effluents*, but now more precisely distinguishes the specific source of the pollutant at Level 3.
**9.2 Garbage & Solid Waste**<br>*municipal waste, manure from livestock operations, mining tailings, litter from cars, agricultural plastics in soil, flotsam & jetsam from boats, microplastics, ghost fishing gear, construction debris, lead from hunting*	Rubbish and other solid materials including those that entangle wildlife.	This category generally is for solid waste outside of designated landfills - landfills themselves should go in *1.2 Commercial & Industrial Areas.* Likewise, toxins leaching from solid waste - for example, mercury leaking out of a landfill into groundwater - should go in *9.1 Water-Borne & Other Effluent Pollution.*	Numbering has changed from 9.4 to 9.2.
**9.3 Air-Borne Pollutants**<br>*acid rain from industry, wind dispersion of pollutants or particulates from farm fields, dust from roads, garbage incineration, methane flairs, smog from vehicle emissions, smoke from forest fires, radioactive fallout*	Atmospheric pollutants from point and nonpoint sources.	It may be difficult to determine the sources of many atmospheric pollutants – and thus hard to take action to counter them.	Numbering has changed from 9.5 to 9.3.
**9.4 Energy Emissions**<br>*beach lights disorienting turtles, heated water from power plants, seismic oil exploration, noise from highways or airplanes, electromagnetic fields from cables, sonar from submarines that disturbs whales, recreational boating wakes*	Inputs of heat, sound, light, or other wave energy that disturb species or ecosystems.	These inputs of energy can have strong effects on some species or ecosystems.	Numbering has changed from 9.6 to 9.4. Edits to the name of the category and definition.
**10. Natural Disasters**	Potentially catastrophic natural disturbances that conservation practitioners may still need to consider, particularly when managing small and/or remnant species populations or ecosystems.	Even though these may be 'natural' system disturbances, nonetheless, if you are charged with conserving a small remnant species population or ecosystem, you may have to take these factors into account in planning and prioritizing threats. For example, if you are managing the last population of Pink Iguanas that live in a volcanic caldera in the Galapagos Islands that could erupt at any moment, or if a large percentage of remaining individuals of a species are in a captive breeding facility that is vulnerable to a hurricane, then clearly you need be concerned about these existential threats to the survival of the species.	Geological events have been demoted one level and put into this new Level 1 order.
**10.1 Geological Events**<br>*volcanic eruptions, earthquakes, tsunamis, avalanches, landslides*	Specific geological events that have potentially catastrophic effects on vulnerable species and ecosystems.		Corresponds to the old Level 1 *10. Geological Events*. Edits to the definition.
**10.2 Severe Weather Events**<br>*rain/wind storms, hurricanes/cyclones/typhoons, hail storms, blizzards, dust storms, floods*	Specific weather events that have potentially catastrophic effecs on vulnerable species and ecosystems.	There is some potential overlap between a specific storm event that would go here and changes to storm regimes which would go in *11.3 Changes in Precipitation & Hydrological Regimes*. But even a 'normal' hurricane or drought can wipe out a small remnant species population. Some floods might be linked to geological events, but we propose to put all floods here.	Corresponds to the old *11.5 Severe / Extreme Weather Events*. Edits to the name of the category.
**11. Climate Change**	Change in climate patterns resulting from increased atmospheric greenhouse gasses.	Strictly speaking individual climatic events may be part of natural disturbance regimes in many ecosystems and are thus technically "stresses" and not "direct threats." But they act as a threat if a species or ecosystem is damaged from other threats and has lost its resilience and is thus vulnerable to the disturbance. In addition, many climatic events (i.e. monsoon or ENSO cycles) may also be increasing in frequency or intensity outside their natural range of variation due to human influences. Even though most projects may not be able to address the root causes of these climate change threats, they do need to consider taking action to counter the effects of these threats. As a general rule, the Conservation Measures Partnership recommends coding the most immediate impacts of climate change as direct threats.	Edits to the definition.
**11.1 Changes in Physical & Chemical Regimes**<br>*ocean acidification, shifting aquatic oxygen minimum zone, changes in salinity, changes in atmospheric CO2 affecting plant growth, loss of sediment, changes in ocean currents, changes in jet stream, changes in cloud cover*	Broad-scale changes in the abiotic conditions of ecosystems.	Primarily includes changes to the geophysical environment including in particular, water and air. Although sea-level rise could potentially be placed here, we have included it under *11.3 Changes in Precipitation & Hydrological Regimes*.	Numbering has changed from 11.2 to 11.1. Edits to the name of the category and definition.
**11.2 Changes in Temperature Regimes**<br>*heat waves, cold spells, freeze/thaw cycles, oceanic temperature changes, marine heat blobs, loss of snowpack or glaciers, melting of sea ice*	Broad-scale changes in temperature mean, variability, seasonality and extremes, including changes in temperature extremes, increased average summer temperature, and decreased minimum winter/spring temperature.	Loss of snowpack and glaciers is often a combination of change in temperature and precipitation regimes, but is assigned here, especially when the issue is the change in temperature above freezing rather than the lack of precipitation.	Numbering has changed from 11.3 to 11.2.
**11.3 Changes in Precipitation & Hydrological Regimes**	Broad-scale changes in precipitation mean, variability, seasonality, and extremes, including	Impacts of specific storm events belong in *10.2 Severe Weather Events.*	Numbering has changed from 11.4 to 11.3

rainfall patterns, droughts, timing of rains, reduced snow accumulation, increased severity of floods, sea-level rise, shrinkage or loss of lakes	variability, seasonality, and extremes, including decreased or increased precipitation, changes in timing of precipitation, changes in form of precipitation (eg snow vs rain), changes in evapotranspiration rates and hydrological cycles, and droughts and floods.		11.4 to 11.3.
**12. Unknown Threats**		Required by IUCN for cases in which the threats to species are unknown.	Added.
