# Supplementary material for: Classification of direct threats to the conservation of ecosystems and species 4.0
Source: Conserv Biol. 2024 Dec 31;39(3):e14434. doi: 10.1111/cobi.14434 (PMC12124163; doi:10.1111/cobi.14434)
Supplement: Supplementary file 4 — Appendix S4. Classification Levels 0‐2 plus Level 3 Types & Modifiers and Level 4 Types [file COBI-39-e14434-s003.pdf]

Table 3b. Classification Levels 0-2 plus Level 3 Types & Modifiers and Level 4 Types

IUCN - CMP Direct Threats Classification v 4.0<br>0 Threat Class<br>L1 L2 (examples, not comprehensive)	Level 3 Type (Beta)	Optional Level 3 Modifiers (Beta)			Level 4 Proposals
**A. Use of Lands & Waters**					
**1. Residential, Commercial & Recreation Areas**					
**1.1 Residential Areas**<br>*urban areas, suburbs, villages, rural housing, vacation homes, shopping areas, offices, schools, hospitals, houses of worship*	Type<br>- Cities & urban areas<br>- Suburbs<br>- Towns, villages & resort housing<br>- Housing outside of municipal / village areas<br>- Other *(describe)*	Lifecycle<br>- Construction *(includes footprint)*<br>- Ongoing (*e.g. bird collisions with houses*<br>- Abandoned	Regulation<br>- Legal & planned/regulated<br>- Legal but not planned/regulated<br>- Illegal		
**1.2 Commercial & Industrial Areas**<br>*stand-alone office parks, stand-alone shopping centers, manufacturing plants, military bases, coal or nuclear power plants, sewage treatment plants, landfills, train yards, ports, airports, land reclamation for industrial development*	Type<br>- Retail / offices<br>- Factories / industrial<br>- Military bases<br>- Power generation<br>- Waste treatment & disposal sites<br>- Transport hubs<br>- Other *(describe)*	Lifecycle<br>- Construction *(includes footprint)*<br>- Ongoing *(e.g. fish entrainment in indust*<br>- Abandoned	Regulation<br>- Legal & planned/regulated<br>- Legal but not planned/regulated<br>- Illegal		
**1.3 Recreation & Tourism Areas**<br>*visitor facilities in parks, campgrounds, football or other sports fields, ski areas, golf courses, beach facilities, fishing piers, marinas, cultural tourist attractions, pilgrimage facilities, cemeteries*	Type<br>- Parks & green spaces<br>- Recreational campgrounds<br>- Sports & athletic fields / ranges<br>- Ski / bike / motor sports areas<br>- Golf courses<br>- Beach facilities<br>- Docks & marinas<br>- Cultural or religous tourism sites<br>- Other *(describe)*	Lifecycle<br>- Construction *(includes footprint)*<br>- Ongoing<br>- Abandoned	Regulation<br>- Legal & planned/regulated<br>- Legal but not planned/regulated<br>- Illegal		
**2. Agriculture & Aquaculture**					
**2.1 Annual & Perennial Non-Timber Crops**<br>*farms, household swidden plots, oil palm plantations, orchards, vineyards, mixed agroforestry systems, biofuel crops, haying practices that disturb nesting birds*	Type<br>- Shifting cultivation<br>- Annual fixed cropping systems<br>- Perennial non-timber cropping systems<br>- Other *(describe)*	Lifecycle<br>- Establishment *(includes footprint)*<br>- Ongoing operations<br>- Abandoned	Regulation<br>- Legal & planned/regulated<br>- Legal but not planned/regulated<br>- Illegal & not planned/regulated	Intensity<br>- Subsistence / artisinal<br>- Small holder<br>- Industrial	Specify the crop(s) being grown and the production system.
**2.2 Wood & Pulp Plantations**<br>*teak, eucalyptus or acacia plantations, pulp or fiber plantations, firewood lots, christmas tree farms*	Type<br>- Timber<br>- Pulpwood<br>- Ornamental trees<br>- Other *(describe)*	Lifecycle<br>- Establishment *(includes footprint)*<br>- Ongoing operations<br>- Abandoned	Regulation<br>- Legal & planned/regulated<br>- Legal but not planned/regulated<br>- Illegal & not planned/regulated	Intensity<br>- Subsistence / artisinal<br>- Small holder<br>- Industrial	
**2.3 Terrestrial Animal Farming, Ranching & Herding**<br>*cattle feed lots, dairy farms, cattle ranching, chicken farms, goat, camel, or yak herding, game farms, bee keeping*	Type<br>- Farming<br>- Ranching<br>- Herding / nomadic<br>- Other *(describe)*	Lifecycle<br>- Establishment *(includes footprint)*<br>- Ongoing operations<br>- Abandoned	Regulation<br>- Legal & planned/regulated<br>- Legal but not planned/regulated<br>- Illegal & not planned/regulated	Intensity<br>- Subsistence / artisinal<br>- Small holder<br>- Industrial	Specify the animal(s) being farmed or ranched and the type of production system.
**2.4 Marine & Freshwater Aquaculture**<br>*fish ponds on farms, shrimp production in artificial ponds, salmon production in pens in natural water, hatchery salmon, seeded shellfish beds, artificial algal beds*	Type<br>- Aquaculture in artificial water bodies *(e.g. fish grown in cor*<br>- Aquaculture in enclosed natural waters *(e.g. fish pens or al*<br>- Aquaculture in open natural waters *(e.g. hatchery salmon r*<br>- Other *(describe)*	Lifecycle<br>- Establishment *(includes footprint)*<br>- Ongoing operations<br>- Abandoned	Regulation<br>- Legal & planned/regulated<br>- Legal but not planned/regulated<br>- Illegal & not planned/regulated	Intensity<br>- Subsistence / artisinal<br>- Small holder<br>- Industrial	Specify the taxa being produced and the type of production system.
**3. Energy Production & Mining**					
**3.1 Oil & Gas Exploration & Extraction**<br>*oil wells, hydraulic fracturing, deep sea natural gas drilling*	Type<br>- Terrestrial drilling<br>- Freshwater or estuarine drilling<br>- Marine drilling<br>- Hydraulic fracturing<br>- Oil sand mining<br>- Other *(describe)*	Lifecycle<br>- Prospecting<br>- Drilling *(includes footprint)*<br>- Ongoing operations<br>- Post-use /abandoned	Ownership / Regulation<br>- Public / govt<br>- Utility company<br>- Corporate<br>- Small holder / private<br>- Illegal	Footprint<br>- Single site<br>- Multiple sites<br>- Diffuse across land/sea scape	
**3.2 Mining & Quarrying**<br>*coal mines, alluvial gold panning, gold mines, rock quarries, sand or salt mining, coral mining, deep sea nodules, guano harvesting*	Type<br>- Underground mines<br>- Open pit mines<br>- Surface removal mining<br>- Surface harvesting *(e.g. peat)*<br>- Alluvial *(e.g. gold panning)*<br>- Ocean mining *(e.g. deep sea nodules)*<br>- Other *(describe)*	Lifecycle<br>- Prospecting<br>- Construction *(includes footprint)*<br>- Ongoing operations<br>- Post-use /abandoned	Ownership / Regulation<br>- Public / govt<br>- Utility company<br>- Corporate<br>- Small holder / private<br>- Illegal	Footprint<br>- Single site<br>- Multiple sites<br>- Diffuse across land/sea scape	Specify the substance(s) being mined.
**3.3 Renewable Energy**<br>*geothermal power production, solar farms, wind farms (including birds or bats flying into windmills), tidal farms*	Type<br>- Wind<br>- Solar *(e.g. photovoltaic panels, concentrators)*<br>- Geothermal<br>- Hydrokinetic *(other than dams which are in 7.2)*<br>- Other *(describe)*	Lifecycle<br>- Construction *(includes footprint)*<br>- Operations *(e.g. bird/bat collisions)*<br>- Post-use / abandoned	Ownership / Regulation<br>- Public / govt<br>- Utility company<br>- Corporate<br>- Small holder / private<br>- Illegal	Footprint<br>- Single site<br>- Multiple sites<br>- Diffuse across land/sea scape	

**4. Transportation, Service & Security Corridors**						
**4.1 Roads, Trails & Railroads**<br>*highways, secondary roads, logging roads, hiking or biking trails, bridges & causeways, vehicle collisions with wildlife, public transport systems, railroads*	Type<br>- Paved highways<br>- Paved secondary roads<br>- Gravel or unpaved roads<br>- Temporary roads *(e.g. on ice)*<br>- Trails<br>- Railroads<br>- Other *(describe)*	Lifecycle<br>- Construction *(includes footprint)*<br>- Operations *(e.g. vehicle collisions)*<br>- Post-use / abandoned	Ownership<br>- Public / gazetted<br>- Industrial (eg logging / ag / mining)<br>- Private &/or informal	Road Size & Use Level (aka Permeability)<br>- Very large/busy = impermeable<br>- Large/busy = selectively permeable<br>- Medium/occasional = permeable<br>- Small/seldom = highly permeable		
**4.2 Utility & Service Lines**<br>*electrical & phone wires, aqueducts, oil & gas pipelines, electrocution of wildlife on powerlines*	Type<br>- Above ground power/data lines<br>- Below ground power/data lines<br>- Commmunications towers *(e.g. cellular, satellite)*<br>- Oil & gas pipelines<br>- Water pipelines / aqueducts<br>- Steam / geothermal pipelines<br>- Other *(describe)*	Lifecycle<br>- Construction *(includes footprint)*<br>- Operations *(e.g. collision, electrocution)*<br>- Post-use / abandoned	Ownership<br>- Public<br>- Industrial *(e.g. logging / ag / mining)*<br>- Private &/or informal	Size / Permeability<br>- Impermeable<br>- Selectively permeable<br>- Permeable<br>- Highly permeable		
**4.3 Shipping Lanes**<br>*maintenance of shipping channels, canals, shipping lanes, ships running into whales, wakes from cargo ships*	Type<br>- Shipping on natural water bodies<br>- Modified shipping lanes *(e.g. dredging, channelization, ice l*<br>- Artificial shipping canals<br>- Other *(describe)*	Lifecycle<br>- Construction (includes footprint)<br>- Operations<br>- Post-use / abandoned	Ownership<br>- Public<br>- Industrial<br>- Private &/or informal	Size & Use Level<br>- Very large/busy = impermeable<br>- Large/busy = selectively permeable<br>- Medium/occasional = permeable<br>- Small/seldom = highly permeable		
**4.4 Atmospheric & Space Activities**<br>*flight paths, jets impacting birds, commercial or military drones, tethered balloons*	Type<br>- Defined flight paths<br>- Distributed flights *(e.g. drones, weather balloons)*<br>- Stationary atmospheric *(internet balloons)*<br>- Space flight<br>- Other *(describe)*	Lifecycle<br>- Construction (includes footprint)<br>- Operations<br>- Post-use / abandoned	Ownership<br>- Public<br>- Industrial<br>- Private &/or informal	Size & Use Level<br>- Very frequent/many<br>- Frequent/many<br>- Occasional/some<br>- Seldom/few		
**4.5 Fencing & Walls**<br>*border walls, fences around farm fields, fences or noise barriers along roads, protected area fencing, disease control fencing*	Type/Purpose<br>- Border walls / security fencing *(targeting humans)*<br>- Domestic animal control fencing<br>- Wildlife control fencing *(keeping animals in or out)*<br>- Aesthetic fencing & noise barriers<br>- Other *(describe)*	Lifecycle<br>- Construction *(includes footprint)*<br>- Operations<br>- Post-use / abandoned	Ownership<br>- Public<br>- Industrial<br>- Private &/or informal	Size / Permeability<br>- High/deep + impermeable<br>- Selectively permeable (eg to small animals or jumpers or has wildlife cross points)<br>- Permeable<br>- Highly permeable		
**B. Use / Management of Species & Ecosystems**						
**5. Biological Resource Use & Control**						
**5.1 Hunting, Collecting & Controlling Terrestrial Animals**<br>*subsistence hunting, collection of feathers or skins used in traditional ceremonies, commercial wild meat hunting, trophy hunting, fur trapping, insect collecting, pet trade, honey or egg collection, persecution of snakes, culling of deer, killing of crop-raiding animals*	Type/Purpose<br>- Subsistence<br>- Cultural / traditional *(e.g. ceremonial feathers or skins)*<br>- Recreational<br>- Commercial<br>- Scientific / educational<br>- Persecution<br>- Control<br>- Other *(describe)*	Intentionality<br>- Targeted species<br>- Incidentally taken species (retained) *(e.g*<br>- Incidentally affected species (not retain<br>- Ecosystem degradation *(e.g. burning for*	Legality (aka IUU-ness) *(multi select)*<br>- Fully legal<br>- Illegal in this instance<br>- Fully illegal *(e.g. CITES Appendix I or nat*<br><br>- Reported<br>- Unreported<br><br>- Regulated<br>- Unregulated	Commercial Trade Level *(UTCS 1.0)*<br>- Local subsistence<br>- Local commercial<br>- National commercial<br>- International commercial	End Use *(UTCS 1.0)*<br>- Food - human<br>- Food - animal<br>- Medicine - human & veterinary<br>- Poisons<br>- Manufacturing chemicals<br>- Other chemicals<br>- Fuels<br>- Fibre<br>- Construction or structural<br>- Wearing apparel, accessories<br>- Other household goods<br>- Handicrafts, jewellery, etc.<br>- Pets/display animals, horticulture<br>- Research<br>- Sport hunting/specimen collecting<br>- Establish ex-situ production*<br>- Other (free text)<br>- Unknown	Specify the taxa being hunted / collected / controlled.
**5.2 Gathering, Harvesting & Controlling Terrestrial Plants & Fungi**<br>*gathering wild fruit, mushrooms, orchids, rattan, lichen, or herbs for traditional medicine, collecting forage for stall fed animals, non-woody biomass harvesting, rubber or maple syrup tapping, control of host plants to combat timber diseases*	Type/Purpose<br>- Subsistence<br>- Cultural / traditional<br>- Recreational<br>- Commercial<br>- Scientific / educational<br>- Persecution *(e.g. removing stinging plants)*<br>- Control<br>- Other (describe)	Intentionality<br>- Targeted species<br>- Incidentally taken species (retained) *(e.g*<br>- Incidentally affected species (not retain<br>- Ecosystem degradation	Legality (aka IUU-ness) *(multi select)*<br>- Fully legal<br>- Illegal in this instance<br>- Fully illegal *(e.g. CITES Appendix I or nat*<br><br>- Reported<br>- Unreported<br><br>- Regulated<br>- Unregulated	Commercial Trade Level *(UTCS 1.0)*<br>- Local subsistence<br>- Local commercial<br>- National commercial<br>- International commercial	End Use *(UTCS 1.0)*<br>- Food - human<br>- Food - animal<br>- Medicine - human & veterinary<br>- Poisons<br>- Manufacturing chemicals<br>- Other chemicals<br>- Fuels<br>- Fibre<br>- Construction or structural<br>- Wearing apparel, accessories<br>- Other household goods<br>- Handicrafts, jewellery, etc.<br>- Pets/display animals, horticulture<br>- Research<br>- Sport hunting/specimen collecting<br>- Establish ex-situ production*<br>- Other (free text)<br>- Unknown	Specify the taxa being gathered / harvested / controlled.

5.3 Logging, Harvesting & Controlling Trees

clear cutting of hardwoods, selective commercial logging of ironwood, pulp operations, woody biomass collection, fuel wood collection, charcoal production, coppicing, tree thinning

Type/Purpose
- Subsistence
- Cultural / traditional
- Recreational
- Commercial
- Scientific / educational
- Persecution
- Control
- Other *(describe)*

Intentionality
- Targeted species
- Incidentally taken species (retained) *(e.g*
- Incidentally affected species (not retain
- Ecosystem degradation *(e.g. bottom tra*

Legality (aka IUU-ness) *(multi select)*
- Fully legal
- Illegal in this instance
- Fully illegal *(e.g. CITES Appendix I or nat*

- Reported
- Unreported

- Regulated
- Unregulated

Commercial Trade Level *(UTCS 1.0)*
- Local subsistence
- Local commercial
- National commercial
- International commercial

End Use *(UTCS 1.0)*
- Food - human
- Food - animal
- Medicine - human & veterinary
- Poisons
- Manufacturing chemicals
- Other chemicals
- Fuels
- Fibre
- Construction or structural
- Wearing apparel, accessories
- Other household goods
- Handicrafts, jewellery, etc.
- Pets/display animals, horticulture
- Research
- Sport hunting/specimen collecting
- Establish ex-situ production*
- Other (free text)
- Unknown

Specify the taxa being logged / harvested / controlled.

5.4 Fishing, Harvesting & Controlling Aquatic Species

net fishing, hook and line fishing, trawling, blast fishing, spear fishing, shellfish harvesting, whaling, seal hunting, turtle egg collection, live coral collection, aquarium fish collection, seaweed collection, persecution of sharks, control of seals

Type/Purpose
- Subsistence
- Cultural / traditional
- Recreational
- Commercial
- Scientific / educational
- Persecution
- Control
- Other *(describe)*

Intentionality
- Targeted species
- Incidentally taken species (retained) *(e.g. turtles caught in shrimp nets that are eaten or sold)*
- Incidentally affected species (not retained) *(e.g. turtles caught in shrimp nets that are thrown back)*
- Ecosystem degradation *(e.g. bottom trawling, coral reef bombing)*

Legality (aka IUU-ness) *(multi select)*
- Fully legal
- Illegal in this instance
- Fully illegal *(e.g. CITES Appendix I or nat*

- Reported
- Unreported

- Regulated
- Unregulated

Commercial Trade Level *(UTCS 1.0)*
- Local subsistence
- Local commercial
- National commercial
- International commercial

End Use *(UTCS 1.0)*
- Food - human
- Food - animal
- Medicine - human & veterinary
- Poisons
- Manufacturing chemicals
- Other chemicals
- Fuels
- Fibre
- Construction or structural
- Wearing apparel, accessories
- Other household goods
- Handicrafts, jewellery, etc.
- Pets/display animals, horticulture
- Research
- Sport hunting/specimen collecting
- Establish ex-situ production*
- Other (free text)
- Unknown

Specify the taxa being fished / harvested / controlled.

Specifiy the fishing gear being used per the International Standard Statistical Classification of Fishing Gear.

6. Human Intrusions & Disturbances

6.1 Recreational Activities

hikers, mountain bikes, horse riding, off-road vehicles, motorboats, jet-skis, snowmobiles, ultralight planes, temporary campsites, caving, rock-climbing, dive boats, whale watching boats, birdwatchers, pets in recreational areas

Type
- Human powered movement *(e.g. hiking, running, mountain*
- Animal powered movement *(e.g. horseback riding, animal p*
- Motor powered movement *(e.g. jet skis, off-road vehicles, s*
- Site use *(e.g. tent or shelter camping, picknicking)*
- Specific ecosystem resource-based recreation *(e.g. rock clim*
- Wildlife watching *(e.g. birding, whale watching, photograp*
- Other *(describe)*

Timing
- Constant
- Seasonal *(e.g. duing breeding season)*
- Occasional or sporadic

Predictability
- Scheduled / managed
- Semi scheduled / predictable
- Random / unpredictable

Legality (multi-select)
- Legal
- Illegal in this instance
- Fully illegal

- Regulated
- Unregulated

Specify the recreational activities.

6.2 Conflict, Civil Unrest & Security Activities

armed conflict, riots, military training exercises, border patrols, peace keeping activities, guerilla camps, abandoned land mines, defoliation, munitions testing

Type
- Conflict or training *(e.g. battles, training exercises)*
- Movements *(e.g. troop movements, border patrols)*
- Site use *(e.g. temporary military or guerilla camps, hunting*
- Ecosystem alterations *(e.g. mine fields, defoliants, trenches,*
- Other *(describe)*

Timing
- Constant
- Seasonal *(e.g. duing breeding season)*
- Occasional or sporadic

Predictability
- Scheduled / managed
- Semi scheduled / predictable
- Random / unpredictable

Legality (multi-select)
- Legal
- Illegal in this instance
- Fully illegal

- Regulated
- Unregulated

6.3 Other Human Disturbances

drug smuggling or human migration through natural areas, livestock rustling, household water collection, non-recreational bathing in rivers, festivals, pilgrimages, species research, archeological research, vandalism

Type
- Movements *(e.g. 'commuting' to farms, drug smuggling)*
- Site use *(e.g. people & dogs on farms, rural household activ*
- Research & conservation work *(species surveys, conservatio*
- Other *(describe)*

Timing
- Constant
- Seasonal *(e.g. duing breeding season)*
- Occasional or sporadic

Predictability
- Scheduled / managed (eg training exerc
- Semi scheduled / predictable
- Random / unpredictable (outright war)

Legality (multi-select)
- Legal
- Illegal in this instance
- Fully illegal

- Regulated
- Unregulated

7. Natural System Management & Modifications

7.1 Fire & Fire Management

fire suppression to protect homes, inappropriate fire management, building of fire breaks, escaped agricultural fires, arson, campfires, fires for hunting

Type
- Creating ecosystem conditions
- Igniting fires
- Extinguishing fires
- Maintaining / managing fires
- Recovering from fires *(replanting, sediment control?)*
- Other *(describe)*

Intentionality
- Intentional, primary *(setting fires to ma*
- Intentional but secondary *(setting fires t*
- Unintentional *(escaped fire from campfi*
- Malicious / illegal *(arson)*

Lifecyle of structures
- Construction *(includes footprint)*
- Operations
- Post-use / abandoned

Predictability of practices
- Scheduled / managed
- Semi scheduled / predictable
- Random / unpredictable

Impact
- Short term suppressor
- Short term increasor
- Long-term suppressor
- Long-term increasor

**7.2 Dams & Water Management / Use**	Type	Intentionality	Lifecycle of structures	Predictability of practices	
dam construction, dam operations, levees and dikes, channelization, highway culverts, adding drains to wetlands for mosquito control, removal of natural beaver dams, encouraging beaver dams, water catchment areas, snow fences, dew harvesting, surface water withdrawals, groundwater pumping, increased humidity from human water use, water desalination, artificial lakes, birds drowning in artificial reservoirs, water treatment plants, adding lime to acid lakes	- Dams and reservoirs<br>- Levees & dikes / channelization<br>- Culverts & drains<br>- Changes to natural barriers *(e.g. removing beaver dams)*<br>- Water, dew & snow catchment<br>- Withdrawals of surface water<br>- Withdrawals of ground water<br>- Water storage tanks<br>- Water treatment & desalination<br>- Other *(describe)*	- Intentional, primary<br>- Intentional but secondary<br>- Unintentional<br>- Malicious / illegal	- Construction *(includes footprint)*<br>- Operations<br>- Post-use / abandoned	- Scheduled / managed<br>- Semi scheduled / predictable<br>- Random / unpredictable	
**7.3 Earth & Sediment Management**	Type	Intentionality	Lifecycle of structures	Predictability of practices	
dune stablization, sediment fencing, shoreline armoring, beach groins, soil pollution remediation, mine reclamation, land reclamation, dredging (except for shipping lanes)	- Earth management<br>- Sediment management<br>- Earth & soil pollution remediation<br>- Other *(describe)*	- Intentional, primary<br>- Intentional, but secondary<br>- Unintentional<br>- Malicious / illegal	- Construction *(includes footprint)*<br>- Operations<br>- Post-use / abandoned	- Scheduled / managed<br>- Semi scheduled / predictable<br>- Random / unpredictable	
**7.4 Weather & Climate Management**	Type	Intentionality	Lifecycle of structures	Predictability of practices	
cloud seeding, frost prevention, iron 'fertilization' in the ocean, releasing reflective particles in the atmosphere, Lagrange point shades, carbon capture (except for infrastructure)	- Weather management<br>- Climate management<br>- Atmopsheric pollution remediation<br>- Other *(describe)*	- Intentional, primary<br>- Intentional, but secondary<br>- Unintentional / accidental<br>- Malicious / illegal	- Construction *(includes footprint)*<br>- Operations<br>- Post-use / abandoned	- Scheduled / managed<br>- Semi scheduled / predictable<br>- Random / unpredictable	
**7.5 Biological System Management**	Type	Intentionality	Lifecycle of structures	Predictability of practices	Specify the ecosystem or species and the associated management practice.
mowing grass, using cattle to mimic natural grazing processs, removal of snags from streams, gating caves, artificial reef creation, assisted migration, bird feeders, electric barriers to stop invasive fish passage	- Management for overall ecosystem<br>- Management for specific taxa<br>- Other *(describe)*	- Intentional, primary<br>- Intentional, but secondary<br>- Unintentional<br>- Malicious / illegal	- Construction *(includes footprint)*<br>- Operations<br>- Post-use / abandoned	- Scheduled / managed<br>- Semi scheduled / predictable<br>- Random / unpredictable	
**7.6 Removing / Reducing Human Management**	Type	Intentionality	Lifecycle of structures	Predictability of practices	
lack of mowing of meadows, cessation of grazing, removal of livestock and hence loss of dung, stopping predator control, reduction or loss of Indigenous management of key ecosystems, ceasing supplemental feeding of condors	- Management for ecosystem<br>- Management for specific taxa<br>- Other *(describe)*	- Intentional, primary (stop flooding field:<br>- Intentional, but secondary (stop floodin<br>- Unintentional (stop flooding fields beca<br>- Malicious / illegal (stop flooding fields ir	- Removal of long-term structure<br>- Removal of abandoned structure	- Reducing scheduled / managed practice<br>- Reducing semi scheduled / predictable practice - Reducing random / unpredictable practice	Specificy the ecosystem or species and the management action being removed.
**C. Additional Sources of Stress**					
**8. Invasive / Other Problematic Species, Genes & Pathogens**					
**8.1 Invasive Non-Native / Alien Species**	Type *(specific name in Level 4)*	Host Ecosystem Type	Status	Purpose / Intentionality	
rats on islands, feral horses, nonferal household pets, feral household pets, zebra mussels, bamboo, introduction of species for biocontrol, stocking exotic fish, ballast water discharge	- Animal<br>- Plant<br>- Fungi<br>- Other *(describe)*<br>- Unknown taxa	- Terrestrial<br>- Freshwater Aquatic<br>- Brackish / Estuarine<br>- Marine	- Current widespread (historical intro)<br>- Currently present (active intro/spread)<br>- Currently patchy/just introduced<br>- Potential	- Deliberately & purposely introduced/spread by humans *(e.g. for hunting, pest control)*<br>- Deliberately, but not purposely introduced/spread by humans *(e.g. released pet pythons)*<br>- Accidentally introduced/spread by humans *(e.g. transported in cargo)*<br>- Naturally introduced/spread	Refer to Global Invasive Species Database (GISD) and/or Linnean classification to specify the organism(s) you are dealing with to the lowest possible classification level (e.g. Family, Genus, Species and/or Subspecies)
**8.2 Problematic Native Species**	Type *(specific name in Level 4)*	Host Ecosystem Type	Status	Intentionality	
overabundant native deer, algal blooms, insect outbreaks	- Animal<br>- Plant<br>- Fungi<br>- Other taxa *(describe)*<br>- Unknown taxa	- Terrestrial<br>- Freshwater Aquatic<br>- Brackish / Estuarine<br>- Marine	- Current widespread (historical intro)<br>- Currently present (active intro/spread)<br>- Currently patchy/just introduced<br>- Potential	- Deliberately & purposely spread/enhanced by humans *(e.g. game fish stocking)*<br>- Deliberately, but not purposely spread/enhanced by humans *(e.g. released pets)*<br>- Accidentally spread/enhanced by humans *(e.g. spread on recreational boats)*<br>- Naturally spread/enhanced	Refer to Linnean classification to specify the specific organism(s) (Family, Genus, Species and/or Subspecies) that you are dealing with. Could also be a ecological type / guild (eg grazers)
**8.3 Introduced Genetic Material**	Type	Host Species Type	Status	Intentionality	
pesticide resistant crops, hatchery salmon breeding with wild fish, domestic cats breeding with wild cats, restoration projects using non-local seed stock, genetically modified insects for biocontrol, genetically modified trees, genetically modified salmon or shellfish	- Hybridization with domesticated / translocated species<br>- Agricultural or aquaculture enhancements *(e.g. genetically*<br>- Native species enhancements *(e.g. drought resistant trees)*<br>- Native species control *(e.g. daughterless male mosquitoes)*<br>- Other *(describe)*	- Animal species<br>- Plant species<br>- Other species<br>- Unknown species *(describe)*	Current widespread (historical intro)<br>Curently present (active intro/spread)<br>Currently patchy/just introduced<br>Potential	Deliberately & purposely introduced/spread by humans *(e.g. gene drive for bio control)*<br>Deliberately, but not purposely introduced/spread by humans *(e.g. hatchery salmon gene hybridization)*<br>Accidentally introduced/spread by humans<br>Naturally introduced/spread	
**8.4 Pathogens**	Type	Host Species Type	Status	Intentionality	
plague affecting rodents, chronic wasting disease affecting cervids, Dutch elm disease or chestnut blight, Chytrid fungus affecting amphibians, seastar wasting disease	- Prion<br>- Viral<br>- Bacterial / arachae<br>- Fungal<br>- Plant<br>- Protozoan<br>- Animal *(e.g. worms)*<br>- Synthetic pathogens<br>- Unknown cause / agent *(describe)*	- Animal species<br>- Plant species<br>- Other species<br>- Unknown species *(describe)*	- Current widespread (native)<br>- Current widespread (historical intro)<br>- Currently present (active intro/spread)<br>- Currently patchy/just introduced<br>- Potential	- Deliberately & purposely introduced/spread by humans<br>- Deliberately, but not purposely introduced/spread by humans<br>- Accidentally introduced/spread by humans<br>- Naturally introduced/spread	Refer to Linnean classification to specify the specific disease organism (Family, Genus, Species and/or Subspecies) and host species that you are dealing with

9. Pollution

9.1 Water-Borne & Other Effluent Pollution

discharge from municipal waste treatment plants, leaking septic systems, untreated sewage, outhouses, fertilizers and pesticides from lawns and golf-courses, toxic chemicals from factories, illegal dumping of chemicals, nutrient loading from fertilizer run-off, herbicide run-off, manure from feedlots, excess nutrients from aquaculture, oil spills from pipelines, leaching from mine tailings, arsenic from gold mining, leakage from fuel tanks, oil or sediment from roads, road salt, erosion from logging operations, toxic chemicals in dredged river sediments

Source Type
- Residental & commercial development effluents *(e.g. sewa*
- Agriculture & aquaculture effluents *(e.g. farms, livestock)*
- Energy production & mining effluents *(e.g. oil spills)*
- Transportation & service corridor effluents *(e.g. road runofj*
- Biological resource use effluents *(e.g. logging)*
- Human intrusions & disturbance effluents *(e.g. military def*
- Natural system management & modification effluents *(e.g.*
- Historical or unknown source effluents
- Other effluents *(describe)*

Pollutant(s) of Concern (multi-select)
- Excess nutrients
- Excess sediment / material
- Salt
- Toxic chemicals
- Oil, gas, fuels
- Bioactive compounds & allergens
- Pathogens
- Greenhouse gasses / ozone
- Radioactivity
- Macroplastics & other solid waste
- Microplastics & other particles
- Other *(describe)*

Reservoir (multi-select)
- Water
- Soil
- Air
- Organisms
- Other *(describe)*

Traceability
- Current or future point source
- Current or future nonpoint source
- Historical

Legality (multi-select)
- Legal
- Illegal in this instance
- Fully illegal

- Regulated
- Unregulated

- Reported
- Unreported

Intentionality
- Deliberate disposal
- Byproduct
- Accidentally released

Specify the toxins.

9.2 Garbage & Solid Waste

municipal waste, manure from livestock operations, mining tailings, litter from cars, agricultural plastics in soil, flotsam & jetsam from boats, microplastics, ghost fishing gear, construction debris, lead from hunting

Source Type
- Residental & commercial development waste *(e.g. municip*
- Agriculture & aquaculture waste *(e.g. manure)*
- Energy production & mining waste *(e.g. mining tailings)*
- Transportation & service corridor waste *(e.g. flotsam from i*
- Biological resource use waste *(e.g. logging slash, ghost fish*
- Human intrusions & disturbance waste *(e.g. lead from hunt*
- Natural system management & modification waste *(e.g. dre*
- Historical or unknown source waste
- Other waste *(describe)*

Pollutant(s) of Concern
- Excess nutrients
- Excess sediment / material
- Salt
- Toxic chemicals / salt
- Oil, gas, fuels
- Bioactive compounds & allergens
- Pathogens
- Greenhouse gasses / ozone
- Radioactivity
- Macroplastics & other solid waste
- Microplastics & other particles
- Other *(describe)*

Reservoir (multi-select)
- Water
- Soil
- Air
- Organisms
- Other *(describe)*

Traceability
- Current or future point source
- Currrent or future nonpoint source
- Historical

Legality (multi-select)
- Legal
- Illegal in this instance
- Fully illegal

- Regulated
- Unregulated

- Reported
- Unreported

Intentionality
- Deliberate disposal
- Byproduct
- Accidentally released

9.3 Air-Borne Pollutants

acid rain from industry, wind dispersion of pollutants or particulates from farm fields, dust from roads, garbage incineration, methane flairs, smog from vehicle emissions, smoke from forest fires, radioactive fallout

Source Type
- Residental & commercial development emissions *(e.g. fact*
- Agriculture & aquaculture emissions *(e.g. smoke from burn*
- Energy production & mining emissions *(e.g. methane flairs)*
- Transportation & service corridor emissions *(e.g. smog or C*
- Biological resource use emissions *(e.g. smoke from forest fi*
- Human intrusions & disturbance emissions *(e.g. chemical d*
- Natural system management & modification emissions *(e.g*
- Historical or unknown source emissions
- Other emissions *(describe)*

Pollutant(s) of Concern
- Excess nutrients
- Excess sediment / material
- Salt
- Toxic chemicals
- Oil, gas, fuels
- Bioactive compounds & allergens
- Pathogens
- Greenhouse gasses / ozone
- Radioactivity
- Macroplastics & other solid waste
- Microplastics & other particles
- Other *(describe)*

Reservoir (multi-select)
- Water
- Soil
- Air
- Organisms
- Other *(describe)*

Traceability
- Current or future point source
- Currrent or future nonpoint source
- Historical

Legality (multi-select)
- Legal
- Illegal in this instance
- Fully illegal

- Regulated
- Unregulated

- Reported
- Unreported

Intentionality
- Deliberate disposal
- Byproduct
- Accidentally released

9.4 Energy Emissions

beach lights disorienting turtles, heated water from power plants, seismic oil exploration, noise from highways or airplanes, electromagnetic fields from cables, sonar from submarines that disturbs whales, recreational boating wakes

Source Type
- Residental & commercial development emissions *(e.g. heat*
- Agriculture & aquaculture emissions *(e.g. aquaculture light*
- Energy production & mining emissions *(e.g. seismic surveys*
- Transportation & service corridor emissions *(e.g. electroma*
- Biological resource use emissions *(e.g. disturbance from gu*
- Human intrusions & disturbance emissions *(e.g. military so*
- Natural system management & modification emissions *(e.g*
- Historical or unknown source emissions
- Other emissions *(describe)*

Pollutant(s) of Concern
- Light
- Noise
- Electro-magnetic
- Wave energy
- Thermal
- Radioactivity
- Other *(describe)*

Reservoir (multi-select)
- Water
- Soil
- Air
- Organisms
- Other *(describe)*

Traceability
- Current or future point source
- Currrent or future nonpoint source
- Historical

Legality (multi-select)
- Legal
- Illegal in this instance
- Fully illegal

- Regulated
- Unregulated

- Reported
- Unreported

Intentionality
- Deliberate disposal
- Byproduct
- Accidentally released

10. Natural Disasters

10.1 Geological Events

volcanic eruptions, earthquakes, tsunamis, avalanches, landslides

Event Type
- Volcanic activity
- Earthquakes
- Tsunamis
- Avalanches / landslides
- Other *(describe)*

10.2 Severe Weather Events

rain/wind storms, hurricanes/cyclones/typhoons, hail storms, blizzards, dust storms, floods

Event Type
- Storms
- Hurricanes / cyclones / typhoons
- Hail storms
- Blizzards
- Dust storms
- Floods
- Other *(describe)*

11. Climate Change

11.1 Changes in Physical & Chemical Regimes

Change Type

Direction & Magnitude of Change

Aspect of Change

ocean acidification, shifting aquatic oxygen minimum zone, changes in salinity, changes in atmospheric CO2 affecting plant growth, loss of sediment, changes in ocean currents, changes in jet stream, changes in cloud cover	- pH<br>- Salinity<br>- Chemical composition *(e.g. O2 levels in water, C02 levels in*<br>- Sediment regimes<br>- Water current & upwelling patterns<br>- Wave & spray patterns<br>- Wind patterns<br>- Cloud cover<br>- Other *(specify)*	- Increase<br>- Extreme increase<br>- Decrease<br>- Extreme decrease<br>- Increase & decrease<br>- Other *(describe)*	- Intensity of events<br>- Frequency of events<br>- Timing of events<br>- Longer term averages / trends<br>- Longer term variability<br>- Longer term phenology patterns<br>- Other *(describe)*
**11.2 Changes in Temperature Regimes**	Change Type	Direction & Magnitude of Change	Aspect of Change
heat waves, cold spells, freeze/thaw cycles, oceanic temperature changes, marine heat blobs, loss of snowpack or glaciers, melting of sea ice	- Temperature regime *(e.g. heat waves, cold spells)*<br>- Ice/snow regime *(e.g. melting ice, snowpack, or permafrost*<br>- Other *(specify)*	- Increase<br>- Extreme increase<br>- Decrease<br>- Extreme decrease<br>- Increase & decrease<br>- Other *(describe)*	- Intensity of events<br>- Frequency of events<br>- Timing of events<br>- Longer term averages / trends<br>- Longer term variability<br>- Longer term phenology patterns<br>- Other *(describe)*
**11.3 Changes in Precipitation & Hydrological Regimes**	Change Type	Direction & Magnitude of Change	Aspect of Change
rainfall patterns, droughts, timing of rains, reduced snow accumulation, increased severity of floods, sea-level rise, shrinkage or loss of lakes	- Precipitation regime<br>- Hydrological regime<br>- Water levels (e.g. sea level rise, shrinking lakes)<br>- Other *(specify)*	- Increase<br>- Extreme increase<br>- Decrease<br>- Extreme decrease<br>- Increase & decrease<br>- Other *(describe)*	- Intensity of events<br>- Frequency of events<br>- Timing of events<br>- Longer term averages / trends<br>- Longer term variability<br>- Longer term phenology patterns<br>- Other *(describe)*

12. Unknown Threats
